# Fine-tuning of macrophage activation using synthetic rocaglate derivatives

**DOI:** 10.1038/srep24409

**Published:** 2016-04-18

**Authors:** Bidisha Bhattacharya, Sujoy Chatterjee, William G. Devine, Lester Kobzik, Aaron B. Beeler, John A. Porco, Igor Kramnik

**Affiliations:** 1 Pulmonary Center, Department of Medicine, Boston University School of Medicine, National Emerging Infectious Diseases Laboratories (NEIDL), Boston University, Boston, MA, 02118, USA; 2 Department of Chemistry, Center for Molecular Discovery (BU-CMD), Boston University, Boston, MA, 02215, USA; 3 Department of Environmental Health, Harvard School of Public Health, Boston, MA, 02115, USA

## Abstract

Drug-resistant bacteria represent a significant global threat. Given the dearth of new antibiotics, host-directed therapies (HDTs) are especially desirable. As IFN-gamma (IFNγ) plays a central role in host resistance to intracellular bacteria, including *Mycobacterium tuberculosis*, we searched for small molecules to augment the IFNγ response in macrophages. Using an interferon-inducible nuclear protein Ipr1 as a biomarker of macrophage activation, we performed a high-throughput screen and identified molecules that synergized with low concentration of IFNγ. Several active compounds belonged to the flavagline (rocaglate) family. In primary macrophages a subset of rocaglates 1) synergized with low concentrations of IFNγ in stimulating expression of a subset of IFN-inducible genes, including a key regulator of the IFNγ network, Irf1; 2) suppressed the expression of inducible nitric oxide synthase and type I IFN and 3) induced autophagy. These compounds may represent a basis for macrophage-directed therapies that fine-tune macrophage effector functions to combat intracellular pathogens and reduce inflammatory tissue damage. These therapies would be especially relevant to fighting drug-resistant pathogens, where improving host immunity may prove to be the ultimate resource.

Antibiotic-resistant bacteria pose a global threat, already producing profound medical and socio-economic impact in both developed and developing nations. Since pathogens acquire drug resistance faster than development of new antibiotics^1^,^2^, alternative approaches to cure infections caused by antibiotic-resistant bacteria are urgently needed. Therefore, efforts to develop host-directed therapies (HDTs) focusing on disease pathogenesis, rather than eradicating pathogens with antibiotics, have been renewed^3^,^4^.

Broadly, HDTs could work either by 1) stimulating beneficial effector mechanisms of host immunity (resistance) or by 2) reducing tissue damage inflicted either directly by a pathogen or by the immune response and inflammation. Several successful macrophage-based screens were focused on identifying compounds that increase control of intracellular bacteria *in vitro* with the idea to use those compounds to boost effector immunity *in vivo*^3^,^5^. Compounds that stimulate autophagy and promote the delivery of intracellular bacteria to lysosomes to be inactivated exemplify this approach^6^. The second group encompasses anti-inflammatory HDTs, such as corticosteroids and inflammatory cytokine inhibitors, many of which, however, suppress effector mechanisms of immunity. Such HDTs should be used only in combination with antibiotics and, therefore, are of limited utility in cases of infections caused by drug-resistant bacteria. An original approach has been pursued by Roca *et al.*, who treated macrophages with high concentrations of TNFα, which induced maximal effector activity against mycobacteria, but also caused macrophage damage and death via a necroptotic pathway. By inhibiting specific mechanisms of TNFα-induced macrophage death in their model, the authors prevented the cytotoxic effect of high concentrations of TNFα, while preserving the resulting increase in microbicidal activity of macrophages^7^.

The IFNγ pathway represents another promising target for HDTs. It is essential for resistance to a broad range of intracellular pathogens, including various mycobacterial species, as convincingly demonstrated both in experimental mouse models and clinical studies^8^,^9^,^10^. In mycobacterial infections, IFN-mediated pathways play dual roles. First, IFNγ primes macrophages for full activation and potentiates production of highly toxic NO and reactive oxygen species (ROS) in response to activating stimuli, such as TNFα or bacterial ligands. It also has been found to reduce inflammation and reduce tissue damage during tuberculosis independently of bacterial control^11^. During chronic infections, bioavailability of IFNγ may be reduced by co-infections (HIV or helminths), circulating IFNγ-neutralizing autoantibodies^12^,^13^,^14^ among other causes. Therefore, recombinant IFNγ and IFNγ-mimetics that activate macrophages by binding to IFNγ receptor were proposed to substitute for the natural ligand^15^,^16^. However, successful intracellular bacterial pathogens have evolved diverse mechanisms not only to suppress the IFNγ production by T cells, but also to down-regulate the responsiveness of macrophages to this cytokine^17^. Besides, sequestration of macrophages and bacteria within chronic lesions such as TB granulomas may also limit delivery of IFNγ. An alternative strategy would be to increase macrophage sensitivity to IFNγ. Hypothetically, small molecules that synergize with low doses of IFNγ may include compounds that mimic the well-documented, but poorly understood, anti-inflammatory activity of IFNγ *in vivo*.

Our previous studies indicate that the *sst1*/IPR1 pathway is one of the mechanisms of host resilience activated by IFNγ. Using a forward genetic analysis in a mouse TB model, we have characterized a genetic locus *sst1* that specifically controls necrotization of TB granulomas, as well as inflammation and necrosis within inflammatory lesions caused by several taxonomically unrelated intracellular bacterial pathogens that reside in macrophages (*Chlamydia pneumoniae*, *Francisella tularensis* and *Listeria monocytogenes*)^18^,^19^,^20^. Using positional cloning, we have identified an interferon-inducible nuclear protein Ipr1/Sp110 within the *sst1* locus and demonstrated that it mediates the *sst1* effect by regulating macrophage activation and death pathways^18^.

Subsequently we have determined that the Ipr1 protein levels in the nuclei of activated macrophages are controlled by IFNγ in a dose-dependent manner. We exploited this relationship to develop a high-throughput screen for small molecules that enhance effect of IFNγ in macrophages. This screen identified the rocaglate family of compounds on the basis of their capacity to significantly increase levels of nuclear GFP-Ipr1 protein in synergy with IFNγ in a reporter macrophage cell line. We used alternative readouts in primary macrophages to confirm and mechanistically dissect the rocaglate synergy with IFNγ.

## Results

### Ipr1 is a nuclear protein regulated by type 1 and type 2 interferons via transcriptional and post-transcriptional mechanisms

**Figure 1:**
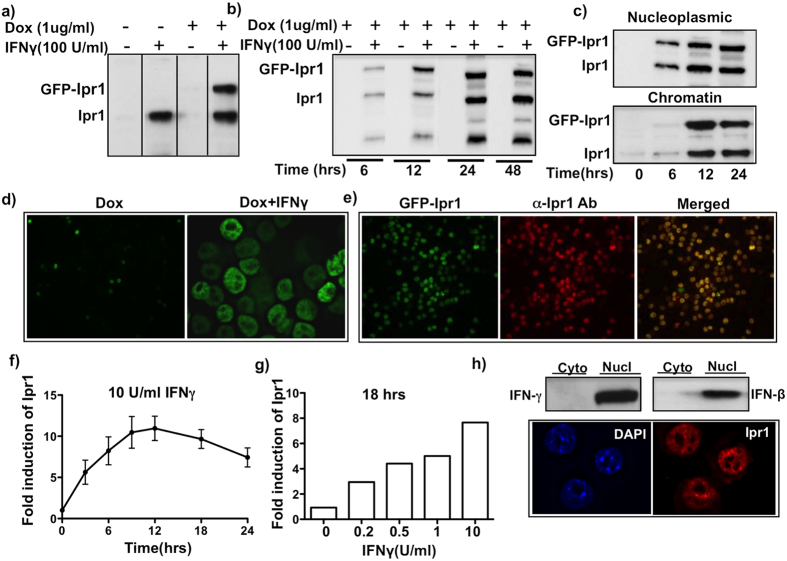
IFNγ regulates expression of Ipr1 at transcriptional and posttranscriptional levels in the nucleus of macrophages. (**a**) Expression of GFP-Ipr1 and endogenous Ipr1 in macrophage cell line J774A.1 clone J7-21. Nuclear extracts were prepared from J7-21 cells untreated (−) or treated with 1 μg/mL dox and/or 100 U/mL IFNγ for 24 hrs. GFP-Ipr1 and endogenous Ipr1 proteins were detected by immunoblotting; (**b**) Nuclear extracts from J7-21 cells treated with 1 μg/mL dox and/or 100 U/mL IFNγ for indicated times were prepared and GFP-Ipr1 expression was detected by immunoblotting. (**c**) Nuclei of J7-21 cells treated with 1 μg/mL dox and 100 U/mL IFNγ for 24 hrs were fractionated into nucleoplasmic and chromatin fractions and endogenous Ipr1 and GFP-Ipr1 was detected by immunoblotting. All immunoblots were carried out using Ipr1 specific rabbit polyclonal antibodies. (**d**) Immunofluorescence of J7-21 cells treated with 1 μg/mL dox alone and in presence of 100 U/mL IFNγ for 24 hrs for GFP-Ipr1 detection (FITC channel). (**e**) J7-21 cells were treated with 1 μg/mL dox and 100 U/mL IFNγ for 24 hrs and stained with Ipr1-specific monoclonal antibody (red, central panel), eGFP-Ipr1 is green (left panel) and merged image is yellow (right panel). (**f**) Real time RT-PCR analysis of the kinetics of Ipr1 mRNA expression in primary macrophages (C57BL/6 J BMDMs) after treatment with10 U/mL IFNγ for indicated times. (**g**) Dose dependent effect of IFNγ on Ipr1 mRNA expression in primary macrophages. B6 BMDMs were treated with indicated doses of IFNγ for 18 hrs. Ipr1 mRNA expression was determined using real-time RT-PCR, normalized to expression of RPS17 mRNA and presented relative to expression in untreated cells (set as 1). All qPCR results represent data from two independent experiments. (**h**) Top panel - Ipr1 protein expression in primary macrophages. Immunoblot analysis of nuclear and cytoplasmic extracts of C57BL/6 BMDM treated with 10 U/mL of IFNγ and 100 U/mL IFNβ for 24 hrs using Ipr1 specific polyclonal antibodies. Immunoblots represent data from at least two independent experiments. Lower panel - Immunofluorescence of B6 BMDMs stimulated with 10 U/mL IFNγ for 24 hrs showing nuclear localization of Ipr1. Cells were stained with anti-Ipr1 monoclonal antibody (red); nuclei are counterstained with DAPI (blue). All microscopic images represent data from at least two independent experiments.

For inducible expression of GFP-tagged Ipr1 fusion protein in the mouse macrophage cell line J774.A1, we used a doxycycline-inducible promoter and lentiviral delivery system as described elsewhere^21^. Clones that displayed no detectable basal GFP and high levels of inducible GFP-Ipr1 expression, were identified using flow cytometry. One of those clones (J7-21) was used for subsequent analyses and assay development. The inducible GFP-Ipr1 expression was confirmed using Ipr1-specific rabbit polyclonal (Fig. 1a and S1) and mouse monoclonal antibodies developed in our laboratory (Fig. 1e,h).

Surprisingly, transcriptional activation of GFP-Ipr1 using doxycycline (Dox) alone was insufficient to induce accumulation of the fusion protein in macrophage nuclei or cytoplasm. Meanwhile, high levels of GFP-Ipr1 and endogenous Ipr1 proteins were detected in the nuclei of macrophages co-treated with Dox (1 μg/mL) and 100 U/mL of IFNγ (Fig. 1a,d). The identity of GFP-Ipr1 in the nuclei was also confirmed using co-staining with Ipr1-specific monoclonal antibody (Fig. 1e). As shown in Fig. 1b, the kinetics and levels of GFP-Ipr1 in the nuclei of IFNγ-activated macrophages paralleled those of the endogenous Ipr1 protein. Both GFP-Ipr1 and endogenous Ipr1 appeared in nucleoplasmic fractions within 6 hrs of macrophage activation with IFNγ and associated with chromatin by 12 hrs (Fig. 1c). To further study association of Ipr1 with chromatin we performed immunoprecipitation of nucleoplasmic and chromatin fractions using GFP-specific antibodies. We found that in the chromatin fraction GFP-Ipr1 associated with heterochromatin in a time-dependent manner (Supplementary Fig. S1).

In primary macrophages, Ipr1 mRNA transcripts were up-regulated at three hours after treatment with IFNγ, peaked at 12 hrs and decreased by 24 hrs, but remained significantly elevated as compared to non-activated macrophages (Fig. 1f). The effect of IFNγ on Ipr1 mRNA expression was dose-dependent (Fig. 1g). The Ipr1 protein levels also increased in the nuclei, but not cytoplasm, of BMDMs within 24 hours of stimulation with type I (IFN-β) or type II (IFNγ) interferons (Fig. 1h, upper panel). Endogenous Ipr1 also associated with chromatin in IFNγ-activated primary macrophages (Fig. 1h, lower panel).

Taken together, these results demonstrate that Ipr1 is an interferon-inducible nuclear protein whose abundance, distribution and stability are regulated by interferons at transcriptional and post-transcriptional levels. Moreover, post-transcriptional regulation of GFP-Ipr1 stability and nuclear localization in J7-21 cells by IFNγ mimics those of the endogenous Ipr1 protein. The association of both proteins with chromatin upon macrophage activation points towards a potential physiological role in chromatin dynamics and/or function induced by interferons.

### The development of a cell-based assay for identifying compounds that synergize with IFNγ based on GFP-Ipr1 nuclear accumulation

**Figure 2:**
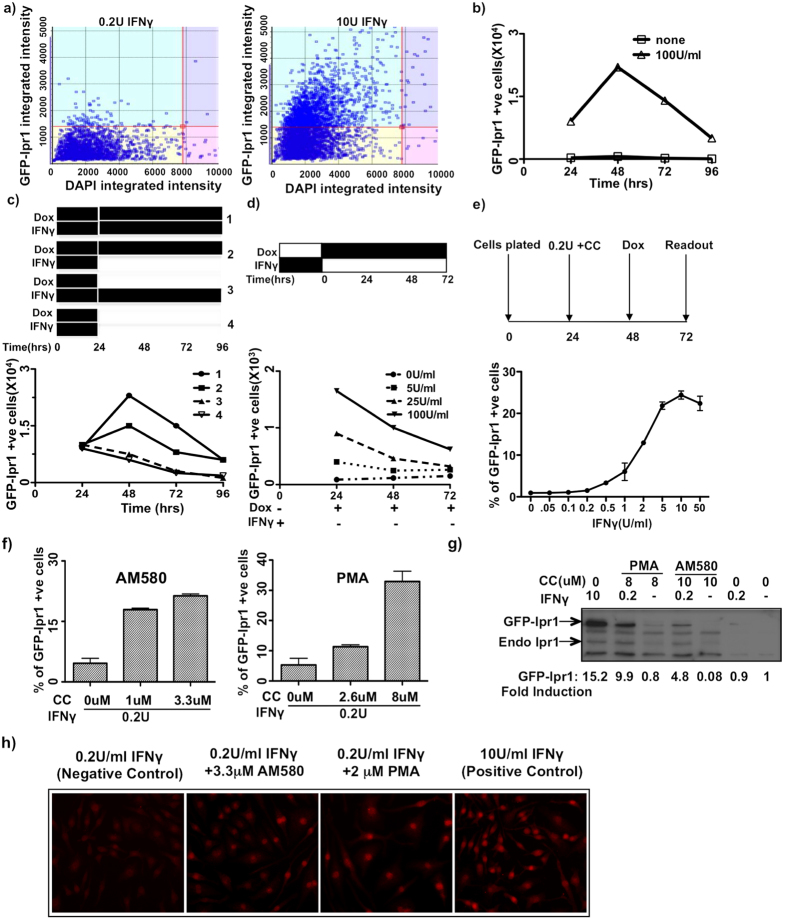
Development and validation of GFP-Ipr1 based screen for small molecules. (**a**) J7-21 cells were treated with 0.2 U and 10 U/mL IFNγ in presence of 1 μg/mL dox for 24 hrs and GFP-Ipr1 expression measured. The gating strategy (mentioned in text) was used to quantify GFP-Ipr1 expression. (**b**) J7-21 cells were treated with 1 μg/mL dox alone (0 U/mL IFNγ) or in presence of 100 U/mL IFNγ for indicated times and fluorescence intensity of GFP-Ipr1 was measured. (**c**) J7-21 cells were primed with 1 μg/mL dox and 100 U/mL IFNγ for 24 hrs and then subjected to the following treatments for additional 72 hrs: 1.dox and IFNγ kept throughout, 2.dox kept and IFNγ removed, 3.dox removed and IFNγ kept, 4.dox and IFNγ removed. GFP-Ipr1 expression was measured using the celigo cytometer. (**d**) J7-21 cells were primed with 5, 25 and 100 U/mL IFNγ for 24 hrs and then removed. 1 μg/mL dox was added and expression of GFP-Ipr1 was measured after 24, 48 and 72 hrs. (**e**) J7-21 cells were treated with different doses of IFNγ for 24 hr. The next day dox(1 μg/mL) was added for 24 hrs and % of cells expressing GFP-Ipr1 was calculated. A schematic representation of the experiment for C, D and E are shown. (**f**) J7-21 cells were treated with different doses of AM580 and PMA in presence of IFNγ(0.2 U/mL) and 1 μg/mL dox and fluorescent intensity of GFP-Ipr1 expression was measured. Cells expressing GFP-Ipr1 was calculated as % expression with respect to 10 U/mL IFNγ. All graphs are representative of at least two independent experiments. (**g**) Immunoblot analysis of whole-cell extracts of J7-21 cells treated with 8 μM PMA and 10 μM AM580 in presence and absence of 0.2 U/mL IFNγ and 1 μg/mL dox for 24 hrs. Blots were probed with Ipr1 polyclonal antibodies. Blots are representative of two independent experiments. Fold induction of GFP-Ipr1 was calculated relative to expression in untreated cells (set as 1) by densitometric analysis after normalizing it to loading control β-actin. (**h**) Microscopy of BMDM from C57BL/6 mice cultured with 2 μM PMA and 3.3 μM AM580 in presence of 0.2 U/mL IFNγ for 24 hrs and stained with Ipr1 monoclonal antibody(red). Images represent data from two independent experiments performed in duplicates.

To develop the screening protocol, we performed time course and dose-response analyses of GFP-Ipr1 expression using an automated cytometry system (Celigo) in a 96-well format. After treatment with IFNγ and Dox, J7-21 cells were fixed, stained with DAPI and analyzed using Celigo on GFP and DAPI channels. GFP fluorescence within nuclear areas delineated by DAPI staining was plotted against total the DAPI signal as presented in Fig. 2a. Gating on the upper left quadrant that contains diploid cells with elevated expression of GFP-Ipr1 in the nuclei was used to normalize the GFP signal against DNA content and provided more specific measure of GFP-Ipr1 induction as compared to total GFP signal. We used this gating strategy to determine thresholds for enumeration of GFP-positive nuclei in subsequent experiments and high throughoutput screening.

Time course analysis demonstrated that the macrophage response induced by maximal concentration of IFNγ (100 U/mL) in our assay peaked at 48 hrs (Fig. 2b). Meanwhile in the nuclear fraction of Dox-treated naive J7-21 cells, we observed the appearance of discrete lower molecular weight bands, also recognized by the Ipr1-specific antibodies (Supplementary Fig. S2), indicating that the Ipr1 protein is cleaved in the nuclei. Next, we determined the requirement of IFNγ and Dox for the GFP-Ipr1 protein nuclear accumulation. The experimental design is outlined schematically in Fig. 2c, upper panel: (Group 1) Dox + IFNγ was kept throughout the course of the experiment; (Group 2) IFNγ was removed after 24 hrs, fresh Dox was added and kept throughout; (Group 3) Dox was removed after 24 hrs and IFNγ kept throughout; (Group 4) both Dox and IFNγ were removed after 24 hrs. GFP-Ipr1 gradually disappeared from the nuclei, if Dox was removed no matter whether IFNγ was present (Group 3), or absent (Group 4). Meanwhile, transient priming with IFNγ for the initial 24 hrs was sufficient to support the nuclear accumulation of GFP-Ipr1 protein for the next 24–48 hrs, if *de novo* synthesis of GFP-Ipr1 was maintained by Dox (Group 2) (Fig. 2c, lower panel). These results demonstrated constant turnover of GFP-Ipr1 in macrophage nuclei and a lasting change conferred by transient IFNγ treatment, presumably at chromatin level that persisted for at least 24 hrs after removal of IFNγ. Next, we treated J7-21 cells with increasing concentrations of IFNγ for 24 hours, removed IFNγ and added Dox (Fig. 2d, upper panel). Nuclear GFP-Ipr1 accumulation was determined using Celigo cytometer at 24 hr intervals (Fig. 2d, lower panel). This experiment demonstrated that after transient stimulation with IFNγ, macrophages retained the ability to accumulate GFP-Ipr1 protein in their nuclei in an IFNγ- dose and time-dependent manner. This observation suggested that the nuclear accumulation of GFP-Ipr1 can be used as a quantitative readout for the IFNγ-primed state of macrophages.

A series of optimization steps for the high-throughput format were performed to minimize handling errors and to attain high well-to-well and plate-to-plate consistency. The finalized screening protocol is presented in Fig. 2e (upper panel): J7-21 cells are primed with IFNγ for 24 hrs; after that Dox (1 μg/mL) is added without washing; and nuclear GFP-Ipr1 protein expression is measured using automated cell cytometer (Celigo) after 24 hrs of Dox addition. In this protocol, stimulation with IFNγ alone produced high sensitivity and linearity in the range of 0.5–10 U/mL of IFNγ (Fig. 2e, lower panel). To screen for compounds that synergize with low doses of IFNγ, we selected 0.2 U/mL of IFNγ - the concentration at which the initial inflexion of GFP-Ipr1 expression was evident in the IFNγ concentration-response curve (Fig. 2e). The J7-21 cells were plated for 24 hrs before priming. First, IFNγ was added to a final concentration of 0.2 U/mL, immediately followed by adding the library of compounds. The cells were incubated for 24 hrs and Dox (1 μg/mL) was added for additional 24 hrs to induce GFP-Ipr1 expression. Thus, the cells were treated with 0.2 U/mL of IFNγ and small molecules for a total of 48 hrs before fixation. After that the nuclei were stained with DAPI and images were acquired using Celigo cytometer. Each compound was tested in triplicate and the wells treated with IFNγ alone at 0.2 U/mL were used to set a threshold and wells treated with 10 U/mL IFNγ served as positive controls. For each compound we calculated percentages of nuclear GFP-Ipr1 positive cells above the threshold (0.2 U/mL of IFNγ alone).

### A pilot study and orthogonal validation

We performed a pilot study using two small molecule libraries: the ICCB library and the LOPAC library. The ICCB library is a collection of 472 diverse biologically active compounds with defined biological activity developed at the Harvard Institute of Chemistry and Cell Biology. The LOPAC (Library of Pharmacologically Active Compounds, Sigma) consists of 1280 compounds with known bioactivity. We identified 24 compounds that increased the expression of GFP-Ipr1 in the presence of 0.2 U/mL IFNγ at least four-fold above the threshold in two independent experiments. Based on their biological properties, we then subdivided these active compounds into two major functional categories: i) compounds that stabilized the GFP-Ipr1 protein by preventing its degradation by proteases (*e.g*. leupeptin and the pan-caspase inhibitor z-VAD) and ii) compounds known to increase macrophage activation directly (several retinoic acid receptor agonists (RAR) and a known protein kinase C activator phorbol myristate acetate (PMA).

We then selected two compounds from the second group; the RAR agonist AM580 and PMA for further assay validation. First, we performed dose-response analyses for these two compounds using the same assay described above (Fig. 2f). Next, we performed orthogonal validation of our assay using Western blot with Ipr1-specific polyclonal rabbit antibodies, which recognized both GFP-Ipr1 and endogenous Ipr1. As shown in Fig. 2g 10 U/mL IFNγ induced the highest expression of both GFP-Ipr1 and endogenous Ipr1 proteins, while at 0.2 U/mL of IFNγ, levels of GFP-Ipr1 and the endogenous Ipr1 proteins were below detection levels. Neither PMA, nor AM580 alone induced Ipr1 expression, as well. However, in J7-21 cells primed with 0.2 U/mL, both PMA and AM580 induced GFP-Ipr1 and endogenous Ipr1 proteins. Next, we tested the ability of PMA and AM580 to synergize with 0.2 U/mL of IFNγ using mouse primary bone marrow-derived macrophages (BMDMs). The Ipr1 protein was detected by immunofluorescence using Ipr1-specific monoclonal antibodies (Fig. 2h). While 0.2 U/mL IFNγ alone did not induce nuclear Ipr1 accumulation, there was a significant increase in nuclear Ipr1 in BMDMs primed with 0.2 U/mL of IFNγ and treated with PMA or AM580. Again, priming with 0.2 U/mL of IFNγ was necessary for Ipr1 induction by either compound. Therefore using orthogonal assays we confirmed synergy between either AM580 or PMA with low dose IFNγ for induction of GFP-Ipr1 and endogenous Ipr1 (both in a macrophage cell line as well as in primary macrophages). These data also pointed towards a plausible mechanism of the Ipr1 posttranscriptional regulation by phosphorylation and proteolytic cleavage. Indeed, Ipr1 protein sequence analysis predicted multiple phosphorylation and protease cleavage sites (Supplementary Fig. S2). We observed stabilization of GFP-Ipr1 in presence of a pan-caspase/cysteine protease inhibitor z-VAD (Supplementary Fig. S2), while **i**n non-activated J7-21 cells GFP-Ipr1 underwent processing into fragments of lower molecular weight (Supplementary Fig. S2). blocking proteases alone with zVAD was insufficient to induce high levels of GFP-Ipr1 and endogenous Ipr1 proteins (Supplementary Fig. S2). We hypothesize that phosphorylation of Ipr1 (induced in our screen by PMA) may mask its protease cleavage sites by causing conformational change and activation-induced protein complex formation (as shown previously)^21^, enabling GFP-Ipr1 translocation to the nucleus, where it dynamically associates with target chromatin via its SAND domain and nuclear matrix via its Sp100 domain. Thus, the accumulation of nuclear chromatin-associated GFP-Ipr1 is likely to be a result of complex signaling and chromatin re-organization processes during macrophage activation. Taken together, these data provided both mechanistic insights and initial validation of our screening strategy.

### Identification of novel rocaglates that work in synergy with IFNγ

**Figure 3:**
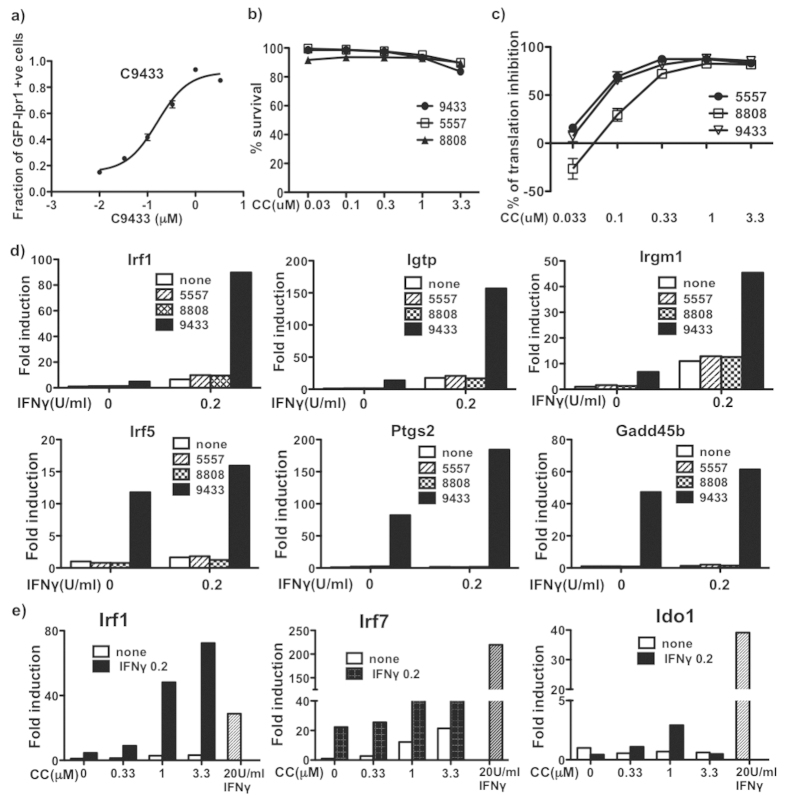
CMLD candidate compound identification and validation. (**a**) Dose response in J774-21 GFP-Ipr1 reporter cell line. J7-21 cells were treated with different doses of C9433 (0.010, 0.033, 0.1, 0.33, 1, 3.3 μM) in the presence of 0.2 U/mL IFNγ for 24 hrs, followed by addition of 1 μg/mL dox for 24 hrs. GFP-Ipr1 expression was measured using automated cytometry. Results are representative of at least two independent experiments performed in triplicates. (**b**) Toxicity of compounds in primary BMDM. BMDM were treated with compounds C9433, C5557 and C8808 at concentrations shown for 24 hrs and % of PI positive cells were calculated. Data is represented as % of survival of two independent experiments performed in duplicates. (**c**) Dose-dependent translation inhibition by rocaglates. 293TR-FLuc cells were treated with rocaglates C9433, C5557 and C8808 (0.033, 0.1, 0.33, 1, 3.3 μM) and luciferase activity was measured after 18 hrs. Two independent experiments were performed in triplicates. (**d**) Comparative effect of 1 μM rocaglates C9433, C5557 and C8808 on gene expression. BMDM were treated with 1 μM compound for 24 hr and the mRNA expression of Irf1, Igtp and Irgm1, Irf5, Gadd45b and Ptgs2 was measured by real-time PCR. (**e**) Effect of C9433 on IFNγ-inducible gene expression. BMDM was treated with different doses of C9433 (0.33, 1, 3.3 μM) in presence and absence of 0.2U IFNγ for 24 hrs and mRNA expression of Irf7, Irf1 and Ido1 was measured by real-time PCR. Cells treated with 20 U/mL IFNγ served as a positive control of gene expression. PCR data are representative of at least two independent experiments.

To identify novel IFNγ-synergistic compounds, we screened a chemical library from the Boston University Center for Molecular Discovery (BU-CMD, www.bu.edu/cmd) which is freely available to biological collaborators. The library consisted of 3840 compounds and is comprised of novel chemotypes rich in structural diversity. Testing the BU-CMD library led to the identification of 30 initial hits; ten of the hits were subsequently confirmed after re-testing in triplicates using quality-controlled compounds from frozen stocks. These candidate compounds (CCs) were subjected to in-depth analyses to exclude assay artifacts and to assess IFNγ-dependence of their activity. First, we tested the candidate compounds in J774 cells that do not express GFP-Ipr1, resulting in exclusion of two DNA intercalating fluorophores with non-specific nuclear fluorescence. Next we tested CCs in J7-21 cells in the presence of IFNγ but in the absence of doxycycline to exclude false positive compounds that would be able to substitute for Dox - none were found. Ultimately, three BU-CMD compounds were selected based on their activity and specificity. All of the top compounds [CMLD005557 (C5557), CMLD008808 (C8808), and CMLD009433 (C9433)] were structurally related derivatives of the natural product rocaglamide A (rocaglates) (see below). Compounds were subjected to quality control (QC) analysis and their activity was confirmed using our initial screening assay. We measured dose-response effects on GFP-Ipr1 expression using the J7-21 clone-based assay of all three CCs in a concentration range 0.03–3.3 μM. The specific activity of C9433 was the highest and dose-dependent with an IC_50_ of 160 nM (Fig. 3a). Toxicity of C5557 and C8808 was higher in comparison to C9433 in J7-21 cells (not shown), but in primary macrophages all three CCs exhibited low toxicity (Fig. 3b). Because rocaglates are known to inhibit protein translation, we compared the effects of C9433, C8808 and C5557 effects on protein biosynthesis using a reporter cell line 293TR-Fluc expressing firefly luciferase under the control of a constitutive promoter^22^. Within the range of concentrations used in our assays, C9433 and C5557 displayed similar translation inhibition activities (IC_50_ values 53 nM and 45 nM respectively) while C8808 was less potent (IC50 value 100 nM), suggesting that specific activities of the CCs in our assay did not strictly parallel their translational inhibition activities (Fig. 3c).

### C9433 modulates IFNγ-driven gene expression in primary macrophages

To further characterize the effects of the rocaglates, we used primary bone marrow-derived macrophages (BMDMs) isolated from C57BL/6 J mice. We compared effects of C9433, C8808 and C5557 at 1 μM (non-toxic concentration, Fig. 3b) on expression of several interferon-inducible genes using qRT-PCR. The combination of C9433 with 0.2 U/mL of IFNγ produced much stronger synergistic effect on Irf1, Igtp and Irgm1 mRNA expression, as compared to C5557 and C8808 (Fig. 3d, upper panels). Next, we compared direct effects of those compounds on expression of genes known to be involved in macrophage differentiation and inflammatory responses. We found that Irf5, Ptgs2 and Gadd45b (MyD118) were induced selectively by C9433, but not with C5557 and C8808 (Fig. 3d, lower panels). These genes are known to participate in control of macrophage differentiation (Irf5^23^ and Gadd45b) and inflammation (Ptgs2)^24^.

To further evaluate the compound C9433, a new batch was synthesized and characterized at the BU-CMD (as described in Supplementary Fig. S3) to assure reproducibility. Next, we compared C9433 and 0.2 U/mL co-stimulation with that of 20 U/mL of IFNγ, the plateau concentration of IFNγ causing changes in gene expression in our assay. We tested expression levels of several IFNγ-inducible genes representing different physiological pathways controlled by IFNγ: Irf1, Irf7, Ido1 (Fig. 3e). Remarkably, the combination of C9433 with 0.2 U/mL of IFNγ produced a very strong synergistic effect on Irf1 mRNA expression, exceeding levels of the Irf1 mRNA induced by 20 U/mL of IFNγ (Fig. 3e). In contrast, the Irf7 and Ido1 mRNA levels induced by the co-stimulation were well below those induced by 20 U/mL of IFNγ. Irf1 is a transcription factor directly induced after IFNγ binds to its specific receptor, and plays a central role in orchestrating macrophage responses to this cytokine^25^. It is essential for host resistance to TB^26^,^27^. Meanwhile, the effects of the IFN-I pathway, which are mediated by Irf7, have been linked to immunosuppression and immunopathology *in vivo*^28^. Therefore, shifting the Irf1/Irf7 balance towards Irf1 using C9433 may boost IFNγ-mediated mechanisms of macrophage resistance and also reduce immunopathology *in vivo*.

### Microarray analysis

To characterize effects of C9433 on macrophage priming with low dose of IFNγ on a global scale, we performed gene expression profiling using Affymetrix Mouse GeneChip 430.2 ST arrays. Comparing BMDMs co-stimulated with C9433 (1 μM) and IFNγ (0.2 U/mL) for 24 hrs to those stimulated with 0.2 U/mL IFNγ alone, we found that 3063 genes were differentially expressed (p < 0.001). Pathway analysis using the Ingenuity package suggested the up-regulation of the Nrf2-mediated oxidative stress response and the Integrin-linked kinase (ILK) pathways. The top down-regulated genes were involved in cholesterol biosynthesis pathways, where the expression of genes encoding two key enzymes in cholesterol biosynthesis DHCR7 and DHCR24 were down-regulated 7.5- and 10-fold respectively. The TGFβ pathway was also found to be down-regulated, consistent with macrophage activation. Among activated pathways relevant to macrophage functions, were those linked to CDKN1 (p21Waf, z-score = 2.86), Vitamin D3 (z-score = 4.5) and Irgm1(z-score = 3.45). Raw microarray data have been deposited in the Gene Expression Omnibus (GEO) as Series GSE72435.

We further explored the C9433 expression signature by using the LINCS1000 tool^29^. After entry of the top 500 up- and down-regulated genes, the program recognized the genes listed in Supplementary Table S1 and performed an expanded version of connectivity map analysis. The results show that the C9433 expression signature is similar to those caused by perturbations that inhibit proteosome (MG132, bortezomib) or protein translation (puromycin, emetine) in the panel of cell lines used in the LINCS1000 platform. Similarly, a comparison of the C9433 expression signature to signatures caused by shRNAs was performed and then sorted to identify the top 50 by percentile rank. This list of targets is also enriched for genes linked to proteasome or protein synthesis functions (Supplementary Table S1). As proteosome inhibition induces proteotoxic stress leading to translation repression, this analysis confirms a known property of other rocaglate compounds - inhibition of translation. Indeed, using a reporter cell line we found that at concentrations used in our assays, C9433 significantly reduced translation (Fig. 3c). In addition, comparison of the C9433 effect on gene expression to signatures caused by over-expression of genes identified 11 genes in the top 10^th^ percentile (i.e. their overexpression caused a similar gene expression profile). Among these genes (BCL10, IFNG, IFNB1, MAGEB6, CDKN1A, TRAF2, CDKN2C, CD40, UGCG, GADD45B, HNF4A, LTBR), IFNG and CDKN1A were also identified in the pathway analysis discussed above. Moreover, we found that C9433 directly induced Gadd45b expression (Fig. 3d), which is consistent with the up-regulation of GADD45B-dependent gene expression signature.

**Figure 4:**
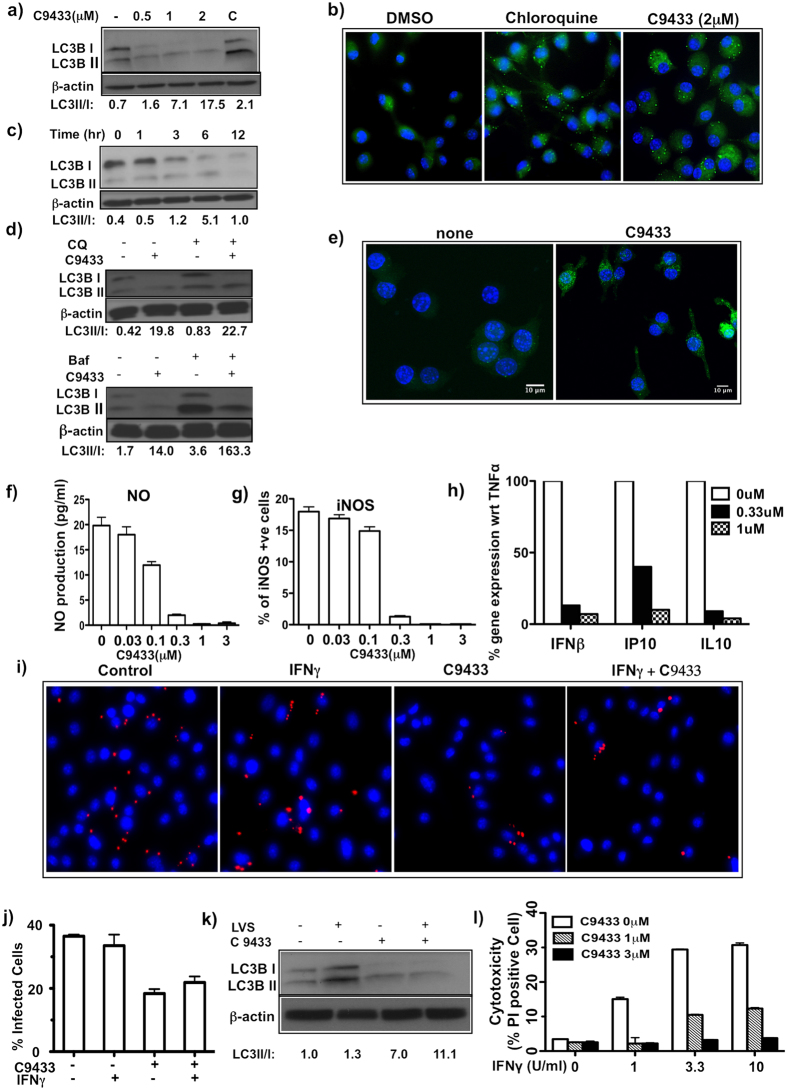
C9433 induces autophagy in primary macrophages and promotes bacterial clearance. (**a**) BMDM were treated with different doses of C9433 for 16 hrs and probed with LC3B mAb. A mix of chloroquine(10 μM) and rapamycin(500 nM) was used as a positive control (C). (**b**) BMDM was treated with compound for 16 hrs and autophagy induction was determined using Cyto-ID Autophagy Detection kit. (**c**) BMDM were treated with 1 μM compound for indicated time and probed with LC3B mAb. (**d**) BMDM were treated with 1 μM compound for 6 hrs. Bafilomycin(50 nM) and chloroquine(30 μM) were added to the cells 2 hrs before harvesting and probed with LC3B antibody. All blots represent data from two independent experiments. (**e**) iBMM cells were treated with 1 μM compound for 6 hrs and autophagic puncta was detected using confocal microscopy. (**f**) BMDM were treated with 15 ng/mL TNFα and 10 U/mL IFNγ for 24 hrs in the presence and absence of C9433 and NO production was determined. (**g**) iNOS expression was determined in the above samples using specific anti-iNOS antibody using celigo cytometer. Two independent experiments performed in triplicates. (**h**) BMDM were treated with 10 ng/mL TNFα in the presence of C9433 for 24 hrs. mRNA expression of IFNβ, IP10 and IL10 were measured by q-PCR. Data is represented as % of gene expression relative to 10 ng/ml TNFα treated cells from two independent experiments. (**i**) BMDMs were either untreated or pretreated with 2 μM C9433, 0.5 U/mL of IFNγ alone or in presence of compound for 16 hrs and then infected with *F.t.* LVS at MOI 1 for 24 hrs. Bacteria were detected using anti-*F.t*. LVS antibodies(red), nuclei counterstained with DAPI(blue). (**j**) 100 cells were counted per condition to detect intracellular bacteria and the % of infected cells were calculated. Microscope images represent data from at least two independent experiments. (**k**) *F.t.* LVS infected BMDM in presence and absence of compound were probed with anti-LC3B mAb. (**l**) BMDM were pretreated with C9433 for 16 hrs in the presence or absence of IFNγ and then infected with *F.t.* LVS at MOI 10. After 7 hrs% of PI- positive cells were enumerated using celigo cytometer. Two independent experiments were performed in triplicates.

In GSEA analysis, the highest positive normalized enrichment score (1.81) was for genes that contained a NF-κB element in their promoters, suggesting that C9433 in combination with IFNγ enhanced NF-κB activity. The expression of NF-κB -inducing kinase (NIK, Map3K14) and TNFα were up-regulated 5-fold suggesting a potential mechanism for NF-κB activation. In addition, up-regulation of a number of stress response genes (ATF4, ATF6, Ddit3, Trib3, Gadd45b, Gadd45g, Hspa1a, and Osgin1) suggested that activation of stress responses might contribute to NF-κB activation by C9433 as well. NF-κB activation may explain superinduction of the Irf1 gene, since it contains a combinatorial NF-κB/GAS element in its promoter^30^. Several other interferon-inducible genes were up-regulated in C9433 plus IFNγ co-stimulated cells (irg1, ifi205, ifit1, ifrd1, igtp). Notably an interferon-inducible negative regulator of IFN-I pathway Usp18 was up-regulated 7.8-fold. We confirmed the C9433-mediated suppression of the IFN-I pathway in an independent assay, where macrophages were stimulated with TNFα (10 ng/mL), a treatment known to induce moderate IFN-I pathway activation^31^. We observed potent suppression of IFNβ, and its downstream targets IP-10 and IL-10 by C9433 (Fig. 4h). Perhaps, the direct suppressive effect of C9433 on the IFN-I pathway may partially account for the sensitization of macrophages to IFNγ,as the two pathways are known to antagonize each other^32^.

We also observed a dramatic upregulation of the prostaglandin E biosynthetic pathway, as the expression of prostaglandin-endoperoxide synthase 2 (Ptgs2, validated using RT-PCR, Fig. 3d) and prostaglandin E synthase (Ptges) containing NF-κB elements in their promoters, increased 16.3 and 3.7-fold respectively, while 15-hydroxy prostaglandin dehydrogenase, which degrades PgE2, was inhibited 34-fold. This coordinated change should lead to increased production of PgE2, which has been recently shown to benefit the host by downregulating the IFN-I pathway^24^ and improved membrane repair in macrophages infected with *M.tb*^11^.

Taken together, our microarray profiling results demonstrated significant reprogramming of macrophage transcriptome by C9433 in combination with 0.2 U/ml of IFNγ that did not simply mimic activation with higher dose of IFNγ, but induced a biased response characterized by relative up-regulation of stress- and NF-κB-responsive genes and suppression of IFN-I pathways.

### C9433 induces autophagy and modulates macrophage effector functions

IFNγ is known to induce two major effector functions relevant for control of intracellular bacteria by macrophages: production of nitric oxide (NO) via activation of inducible NO synthase (iNOS) and induction of phagosome maturation and lysosome fusion via induction of autophagy.

As a general mechanism of cell survival under stress, macrophage autophagy has been also implicated in killing intracellular bacteria via stimulating trafficking of phagocytic vacuoles and promoting phagosome - lysosome fusion^33^,^34^,^35^,^36^. We noted that several interferon-inducible genes up-regulated by C9433 are also regulators of autophagy (Irgm1 in Fig. 3d, Atg7). Therefore, we tested whether C9433 induced autophagy in BMDMs.

During autophagy LC3B-I (Atg8) is converted to LC3B-II through lipidation and inserts into the membrane of autophagosomes. Then, autophagosomes fuse with lysosomes where LC3B-II is degraded by lysosomal hydrolases. The relative increase of LC3B-II and accumulation of LC3-II positive autophagosomes signifies active autophagic flux. Using Western blot analysis with LC3B (Atg8)-specific antibodies, we observed that C9433 (0.5–2 μM) induced the LC3B-I to LC3B-II conversion in a dose-dependent manner (Fig. 4a). To further verify the induction of autophagy, we used a cationic amphiphilic tracer (CAT) that selectively labels autophagic vacuoles (CYTO-ID Autophagy Detection Kit, Enzo Life Sciences). Indeed, treatment of BMDMs with C9433 resulted in accumulation of the CAT-positive vacuoles (Fig. 4b). In a time-course experiment, a maximal LC3-II/LC3-I ratio was observed after 6 hrs of C9433 treatment, which was followed by a decline of total LC3B (Fig. 4c). We demonstrated that LC3B depletion at 12 hrs reflected increased autophagic flux by using inhibitors of lysosomal acidification and protein degradation, chloroquine and bafilomycin A1. Both inhibitors delayed lysosomal degradation of LC3B-II, but not LC3B-I, thus confirming the increase in LC3B-I to LC3B-II transition (Fig. 4d).

We further confirmed autophagy induction by C9433 using an immortalized macrophage cell line, which constitutively expressed eGFP-LC3 fusion protein. As shown in Fig. 4e, eGFP-LC3 fluorescence was largely diffuse in untreated cells with few puncta denoting basal autophagosome formation. Within six hours of exposure to C9433, both eGFP-LC3 puncta-positive cells and the number of puncta per cell significantly increased demonstrating autophagy activation. When autophagosomes fuse with lysosomes and eGFP-LC3II is degraded by lysosomal hydrolases, the LC3-II moiety is degraded faster than the more stable eGFP moiety, resulting in a transient accumulation of eGFP, which serves as an additional confirmation of phagocytic flux. Indeed, we observed a time-dependent accumulation of eGFP by C9433 treatment of the macrophage cell line using Western blot with GFP-specific antibodies (not shown).

Although IFNγ is a weak inducer of iNOS pathway, pretreatment with IFNγ potently primes macrophages for activation of iNOS expression and NO production by TNFα or bacterial ligands^37^. To test whether C9433 can synergize with low doses of IFNγ in priming macrophages for subsequent iNOS activation, we assessed its effect on iNOS expression following priming with IFNγ and stimulation with TNFα. At sub-threshold concentrations, isolated effects of each cytokine on NO production and iNOS mRNA and protein expression were minimal, but co-stimulation with IFNγ and TNFα produced strong synergistic effect on the induction of both iNOS mRNA and protein expression. Unexpectedly, we observed a potent suppressive effect of C9433 on NO production and iNOS expression in BMDM co-stimulated with TNFα and IFNγ. Even when we increased the IFNγ concentration to a standard stimulatory level (10 U/mL), C9433 potently inhibited the IFNγ priming effect on NO production (Fig. 4f). We confirmed this suppressor effect by immunostaining of iNOS protein using automated cytometry (Fig. 4g) and microscopy (Supplementary Fig. S4).

Since C9433 suppressed NO production, one of the key macrophage effector mechanisms, we tested whether C9433 also compromised bacterial control after macrophage infection with intracellular bacteria *Francisella tularensis* Live Vaccine Strain (*F. t.* LVS) *in vitro.* Priming BMDM with IFNγ effectively increases the control of *F. t.* LVS by human and mouse macrophages in a dose-dependent manner^38^,^39^. At 0.5 U/mL the effect of IFNγ on *F. t*. LVS replication in our model was minimal. Therefore, we treated BMDMs with IFNγ (0.5 U/mL), C9433, or a combination of both. After 18 hrs of priming, macrophages were infected at a multiplicity of infection (MOI) of 1 or 10 bacteria per macrophage. Pretreatment of macrophages with C9433 either alone or in combination with 0.5 U/mL of IFNγ did not increase but actually slightly decreased the number of intracellular bacteria per macrophage and the number of infected cells at 24 hrs post infection (Fig. 4i, j). These results suggest that although C9433 suppresses NO pathway, it does not compromise the overall bacterial control by macrophages. It remains to be determined whether treatment with rocaglates helped control the bacteria in NO-independent manner via autophagy induction. Indeed, autophagy induced by *F. tularensis* in infected cells was shown to support bacterial replication by providing energy source to the bacteria^40^. However, we pretreated macrophages with C9433 prior to the infection, which induced autophagic flux earlier and more potently as compared to the infection alone (Fig. 4k). In these settings autophagy may stimulate host cell protective mechanisms, as well.

Infection with intracellular bacteria also causes membrane damage and temporal permeabilization of macrophage membranes within hours^41^. Membrane damage is repaired in autophagy- and PgE2-dependent manner. Since C9433 stimulates both pathways, we wondered whether it might also prevent or reduce the membrane damage induced by the *F. t.* LVS infection. We observed that *F. t.* LVS infection induced membrane permeabilization (PI positive cells) of IFNγ-primed macrophages within 7 hrs of infection (Fig. 4l). However, macrophages primed with IFNγ in the presence of C9433 had a significantly smaller fraction of PI-positive cells, suggesting that pretreatment with C9433 either reduced the membrane damage or enhanced the membrane repair.

### A subset of rocaglates mimics the effects of C9433

**Figure 5:**
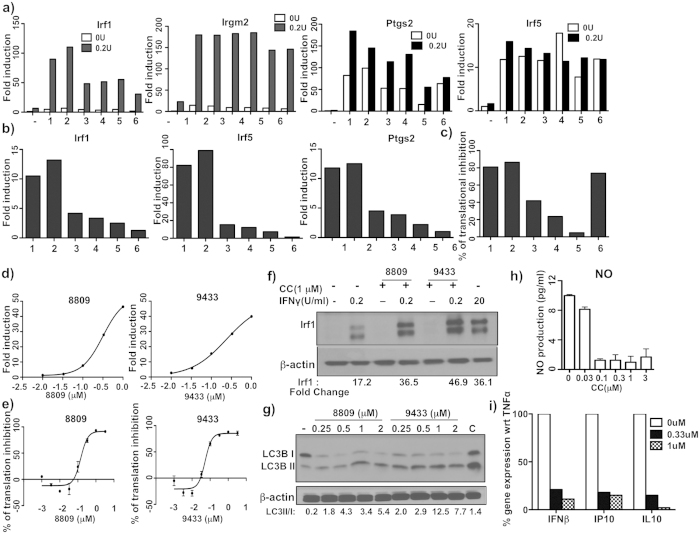
Comparative activity and characterization of new rocaglates. (**a**) BMDM were treated with 1 μM compound (1-C9433, 2-C8809, 3-C10021, 4-C7564, 5-C7565, and 6-C10361) alone and in presence of 0.2 U/mL IFNγ for 24 hr. mRNA expression of Irf1, Irgm2, Irf5 and Ptgs2 was measured by qPCR. Data is presented relative to expression in untreated cells (set as 1). (**b**) BMDM were treated with 0.33 μM compounds (mentioned above) in presence and/or absence of 0.2 U/mL IFNγ for 24 hr. mRNA expression of Irf1, Irf5 and Ptgs2 was measured by qPCR. Data is presented relative to expression in 0.2U IFNγ treated cells for Irf1 and untreated for Irf5 and Ptgs2 (set as 1). (**c**) 293TR-FLuc cells were treated with 0.33 μM compounds (mentioned above) and luciferase activity was measured after 18 hrs. Data represents values from two independent experiments. (**d**) BMDM were treated with different doses of C9433 and C8809 in presence of 0.2 U/mL IFNγ for 24 hrs. mRNA expression of Irf1 was measured by qPCR. Data is presented relative to expression in 0.2U IFNγ treated cells (set as 1). (**e**) 293 T-FLuc cells were treated with different doses of C9433 and C8809 and the luciferase activity was measured after 18 hrs in triplicates. (**f**) BMDM were treated with C8809 and C9433 for 24 hrs in the presence and absence of 0.2 U/mL IFNγ and IRF1 induction was detected by immunoblotting. 20 U/mL IFNγ treated cells served as positive controls. (**g**) BMDM were treated with different doses of C8809 and C9433 for 6 hrs and probed with anti-LC3B mAb. A mix of chloroquine(10 μM) and rapamycin(500 nM) served as positive control (C). All immunoblots are representative of at least two independent experiments. (**h**) BMDM were treated with 15 ng/mL TNFα and 10 U/mL IFNγ for 24 hrs in the presence of different doses of C8809 and NO production was measured in triplicates. (**i**) BMDM were treated with 10 ng/ml TNFα in the presence of C8809 for 24 hrs. mRNA expression of IFNβ, IP10 and IL10 were measured by q-PCR. Data is calculated as % of gene expression relative to 10 ng/mL TNFα treated cells. All q-PCR results were normalized to expression of 18S and are representative of at least two independent experiments.

We screened an additional library of 69 rocaglate derivatives from the BU-CMD collection and identified 5 novel compounds, whose activity in primary macrophages was similar to C9433: at 1 μM they synergized with 0.2 U/mL of IFNγ as demonstrated by Irf1 and Irgm2 mRNA induction (Fig. 5a), and directly induced Irf5 and Ptgs2 mRNA expression (Fig. 5a). Next we ranked their synergistic activity at a lower concentration (0.3 μM): C8809 (#2) was the most active, while 10361 (#6) was the least active (Fig. 5b and Supplementary Fig. S5). However, the translation inhibition effect of C9433, C8809, and C10361 (rocaglaol, a derivative of rocaglamide A) at 0.3 μM was similar (Fig. 5c). Thus, the effect of rocaglates on gene expression did not completely parallel their inhibitory activity on protein translation.

Since C8809 was the most active among the newly identified rocaglates, we compared its dose-dependent effects to that of C9433 on translation inhibition and Irf1 gene expression. While C9433 was a more potent translation inhibitor compared to C8809 with EC_50_ values of 53 nM and 140 nM, respectively (Fig. 5e), both compounds had similar EC_50_ values in the Irf1 gene expression assay −240 nM and 320 nM, respectively (Fig. 5d). Importantly, both compounds also induced IRF1 protein expression strictly in cooperation with 0.2 U/mL of IFNγ to levels similar to those induced by IFNγ alone at 20 U/mL(Fig. 5f).

Similar autophagy-inducing activities of C9433 and C8809 were demonstrated using Western blot analysis, which showed a dose-dependent increase in the expression of LC3BII protein (Fig. 5g), and confirmed using an immortalized macrophage cell line expressing eGFP-LC3 fusion protein (data not shown). Finally, C8809 suppressed both NO production and iNOS expression in primary macrophages co-stimulated with TNFα and IFNγ in a dose-dependent manner (Fig. 5h and Supplementary Fig. S4), as well as the type I IFN pathway activation by TNFα(Fig. 5i). Thus, functional effects of C8809 and C9433 in macrophages substantially overlapped.

### Translation inhibition by rocaglates is necessary for gene expression induction in primary macrophages

**Figure 6:**
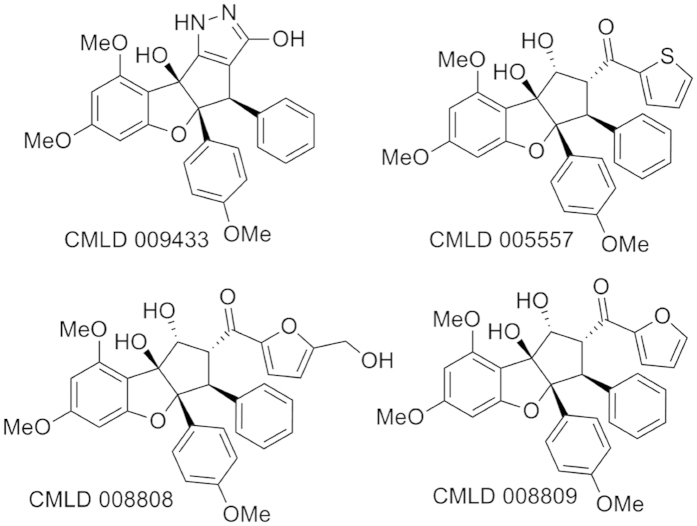
Chemical structures of rocaglates used in the study.

All rocaglates identified in our primary screen (C9433, C8808, C5557 and C8809, Fig. 6) inhibited protein translation (Figs 3c and 5c), suppressed NO production and induced autophagy (the C8808 and C5557 data are presented in Supplementary Fig. S6). We concluded that the autophagy induction and NO pathway inhibition by rocaglates are mechanistically linked to their well-established translation inhibition activity. In this scenario, the translational inhibition may directly lead to autophagy, which, in turn, would inhibit NO production, as described in the literature. However, these data also demonstrated that translation inhibition was not sufficient to account for the unique property of some rocaglates (most notably C9433 and C8809) to synergize with IFNγ in macrophage activation, as determined using gene expression analyses.

**Figure 7:**
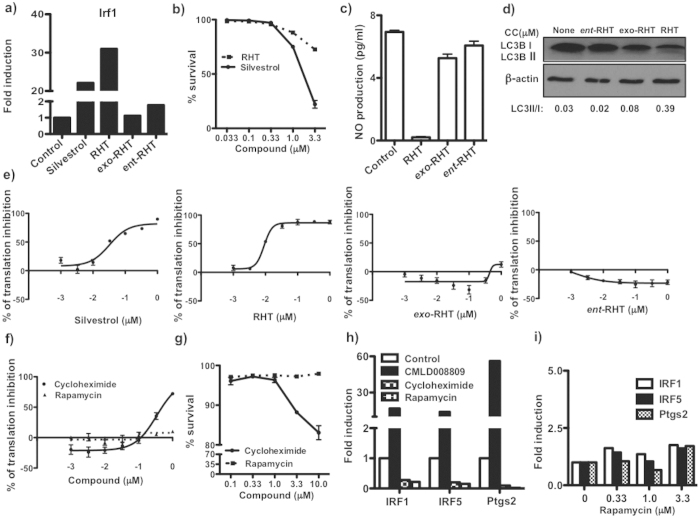
Comaparative activity of known rocaglates and translational inhibitors. (**a**) BMDM were treated with 0.33 μM compounds in presence of 0.2 U/mL IFNγ for 24 hr. mRNA expression of Irf1 was measured by qPCR and normalized to expression of 18 S. Data is presented relative to expression in 0.2U IFNγ treated cells (set as 1) and represents results from two independent experiments. (**b**) BMDM were treated with silvestrol and RHT at concentrations shown for 24 hrs and % of PI positive cells were calculated. Data is represented as % of survival of two independent experiments performed in duplicates. (**c**) BMDM were treated with 15 ng/mL TNFα and 10 U/mL IFNγ for 24 hrs in the presence and absence of 1 μM compounds and production of NO (assayed as NO_2_^−^) was determined. All measurements for NO production were performed in triplicates. (**d**) BMDM were treated with 1 μM compounds for 6 hrs and autophagy was determined by increase of LC3B-II to LC3B-I ratio by immunoblotting. Blots represent data of two independent experiments. 293 T-FLuc cells were treated with different doses of (**e**) silvestrol, RHT, exo-RHT and *ent*-RHT and (**f**) cycloheximide and rapamycin. The luciferase activity was measured after 18 hrs. Data represents values from experiment performed in triplicates. (**g**) BMDM were treated with cycloheximide and rapamycin at concentrations shown for 24 hrs and % of PI positive cells were calculated. Data is represented as % of survival of two independent experiments performed in duplicates. (**h**) BMDM were treated with 0.33 μM compounds in presence of 0.2 U/mL IFNγ for 24 hr. mRNA expression of Irf1, Irf5 and Ptgs2 was measured by qPCR and normalized to expression of 18 S. Data is presented relative to expression in 0.2U IFNγ treated cells (set as 1) and represents results from two independent experiments. (**i**) BMDM were treated with 0.33, 1 and 3.3 μM rapamycin in presence of 0.2 U/mL IFNγ for 24 hr. mRNA expression of Irf1, Irf5 and Ptgs2 was measured by qPCR and normalized to expression of 18S. Data is presented relative to expression in 0.2U IFNγ treated cells (set as 1) and represents results from two independent experiments.

In order to determine whether translation inhibition was necessary, we tested silvestrol and rohitinib (RHT), two well-studied rocaglates possessing both potent translation inhibition and cytotoxic activities. Both are known to suppress the cap-dependent translation initiation complex eIF4F by binding to and inhibiting an RNA helicase (eIF4A)^22^,^42^,^43^. Both RHT and silvestrol showed significant toxicity at the highest two doses (1 and 3.3 μM) in primary macrophages (Fig. 7b) and potent translation inhibition (EC_50_ for silvestrol is 40 nM, for RHT it is 10 nM, Fig. 7e). Notably silvestrol was more toxic to primary macrophages, but was found to be less active in comparison to RHT. At 0.33 μM, the highest concentration that was not cytotoxic for primary macrophages within 24 hrs, both compounds synergized with IFNγ to induce Irf1 mRNA expression (Fig. 7a).

Next we compared activities of RHT and its isomers which are inactive as translation inhibitors (Fig. 7e) *exo*-RHT (*exo*-stereoisomer, CMLD10401) and *ent*-RHT (inactive enantiomer, CMLD10531)^43^,^44^(see Supplementary Fig. S7 for chemical structures). Neither *exo*-RHT nor *ent*-RHT stimulated Irf1 expression (Fig. 7a), inhibited NO production (Fig. 7c), nor induced autophagy (Fig. 7d). These data demonstrate that all of the above rocaglate activities, including synergy with IFNγ in gene expression, are linked to their ability to inhibit translation initiation. However, the known translation elongation inhibitor cycloheximide (EC_50_ is 300 nM, Fig. 7f) completely failed to stimulate Irf1, Irf5 and Ptgs2 gene expression in macrophages (Fig. 7h). Note, the cycloheximide and RHT toxicities in our model were similar (Fig.7b,g). Rapamycin is a suppressor of cap-dependent translation known to induce autophagy via inhibition of the mTOR pathway, in an eIF4A-independent manner. It also failed to stimulate the macrophage gene expression even at concentrations up to 3.3 μM (Fig. 7i), which still were not toxic for primary macrophages (Fig. 7g). Taken together, these data demonstrate that stimulation of gene expression in synergy with IFNγ by rocaglates is tightly associated with their activity to inhibit protein translation.

## Discussion

An unexpected outcome of these studies was the discovery of a unique property of a subset of rocaglates to synergize with IFNγ in macrophage activation in addition to a more broadly distributed abilities of rocaglates to block protein translation and to induce stress response and autophagy in an IFNγ-independent manner.

Rocaglates and rocaglamide derivatives are cyclopenta-[*b*]benzofurans with unusual carbon skeletons found in the plant genus *Aglaia* which is indigenous to Southeast Asia. These compounds have been used in traditional Chinese medicine. Antileukemic and/or cytotoxic activity of certain rocaglamide derivatives has been reported^34^,^45^. Silvestrol (isolated from *Aglaia foveolata*) is a potent inhibitor of protein synthesis and has cytotoxic activity similar to many FDA-approved anticancer agents. More potent synthetic rocaglates are being developed for cancer therapy based on their ability to target the malignant anabolic state^22^ by inhibiting cap-dependent protein translation and HSF1 activity. In addition, rocaglamides have been shown to bind prohibitins, and suppress MEK-ERK signaling and cell cycle progression in malignant cells^46^.

Notably, it was shown that rocaglamide and its derivatives represent highly potent and specific inhibitors of TNFα-R or PMA-induced NF-κB activity in different mouse and human T cell lines^47^. A synthetic derivative of rocaglaol was able to reduce tissue inflammation and neuronal cell death *in vivo* by inhibiting NF-κB and AP-1 signaling, resulting in significant neuroprotection in animal models of neurodegeneration^48^. In addition, some rocaglamide derivatives have been suggested as a new source of NF-AT specific inhibitors for the treatment of certain inflammatory diseases^49^. Currently it is unknown whether biological properties of rocaglamide derivatives may be explained by a common mechanism, such as inhibition of cap-dependent translation or several pathways are being engaged simultaneously or selectively.

Here in we report a novel activity of rocaglates- a synergistic effect with low doses of IFNγ on activation of Irf1 mRNA expression in primary (non-transformed) macrophages. IRF1 protein is a major transcription factor in the IFNγ-regulated network^50^, which is essential for host resistance to TB and other intracellular pathogens^51^,^52^,^53^. Its up-regulation may lead to global macrophage reprogramming beneficial for host resistance to intracellular pathogens.

Considering possible mechanisms of synergy between a rocaglate and IFNγ, we envision several scenarios based on rocaglate-mediated translational inhibition. First, the Irf1 gene is a direct transcriptional target of IFNγ, based on the gamma-activated site (GAS) site element in its promoter, an element that overlaps with a NF-κB motif^30^. The overlap accounts for synergistic interactions of IFNγ and NF-κB pathways in the control of Irf1 gene expression. The NF-κB pathway can be activated by translation inhibition directly by changing stoichiometry of activating and inhibiting subunits^54^. This effect is much less potent as compared to NF-κB activation induced via TNFα-R. However, subthreshold activation of NF-κB by a rocaglate in combination with low dose of IFNγ may produce synergistic effect on Irf1 due to the overlapping GAS/NF-κB elements in its promoter. Second, translation inhibitors were shown to induce activation of stress kinases, including JNK, that activate the AP-1 transcription complex, which may synergize with STAT1 on the Irf1 promoter^55^,^56^.

Another, more selective, mechanism may be explained by the ability of rocaglates to specifically inhibit cap-dependent protein translation by binding to and inhibiting the RNA helicase eIF4A. Thus, they do not block protein translation in general, but induce a switch from cap-dependent to cap-independent translation. This switch plays a physiological role in cell adaptation to stress conditions, where cap-dependent translation is repressed because of eIF2α phosphorylation by stress kinases. For example, control of translation via protein-regulated upstream open reading frames (uORFs) that inhibit translation initiation of stress-responsive downstream ORFs has been demonstrated^57^. Blocking the cap-dependent uORF translation up-regulates translation of a set of stress-responsive proteins^58^. Also, many proteins involved in stress responses and survival are translated in cap-independent manner using internal ribosome entry sites (IRES)^59^. The blockade of cap-dependent translation may serve an especially important signaling role in macrophages that, as a rule, must adapt to and perform their functions in particularly stressful environments.

Interestingly IFNγ itself regulates protein translation via up-regulation and activation of PKR, subsequent eIF2α phosphorylation and translational repression^60^,^61^. Meanwhile, a pool of interferon-stimulated proteins increases^62^. Perhaps, rocaglate-induced inhibition of cap-dependent translation potentiates this effect of IFNγ, which shifts translational activity towards cap-independent translation *via* uORF and IRES-dependent mechanisms leading to remodeling of the macrophage proteome more towards stress- and activation-induced pattern. Thus, inhibition of cap-dependent translation by IFNγ and rocaglates may mimic stress and induce stress response proteins to facilitate macrophage pre-adaptation to imminent stress and, thus, increase their resilience to subsequent challenges.

Importantly, not all proteins are equally sensitive to inhibition of eIF4A helicase activity. A recent study identified the hallmarks of eIF4A-dependent transcripts using transcriptome-scale ribosome footprinting. These include specific UTR sequences that can form complex RNA G-quadruplex structures^63^. Thus, the helicase dependence of protein translation is necessitated by the presence of mRNA tertiary structures in specific transcripts. This model suggests that compounds that quantitatively differ in eIF4A RNA helicase inhibition may differentially affect protein subsets. Notably, among the most eIF4A-dependent and rocaglate-sensitive transcripts were a number of oncogenes, superenhancer-associated transcription factors, and epigenetic regulators. Conceivably, some rocaglates may synergize with IFNγ in macrophages by blocking translation of a particular subset of proteins that oppose macrophage activation, such as growth factor-induced oncogenes for example. Therefore, use of rocaglate derivatives with varying inhibitory activities may serve to fine tune cell responses in a cell type-and activation-specific manner. The novel rocaglates identified in this study are overall weaker translation inhibitors as compared to RHT and silvestrol, the later being known as powerful inhibitors of translation which are cytotoxic to leukemic cells. Accordingly, the new rocaglates are less toxic in primary macrophages and their effect on macrophage gene expression may be more selective. There is also a possibility that a subset of rocaglates may target other RNA/DEAD Box helicases in macrophages in addition to eIF4A. Attempts to identify potential new targets and pathways of rocaglate compounds that may synergize with IFNγ in macrophage activation are underway in our laboratories.

Taken together our studies have revealed a novel property of a subset of rocaglates to synergize with IFNγ in macrophage activation, which may lead to the development of macrophage-directed therapies that target specific aspects of macrophage activation. The ultimate goal of these therapies would be to reduce inflammatory tissue damage *in vivo* by improving macrophage resilience and fine-tuning their effector functions for specific infections and inflammatory conditions.

## Materials and Methods

### Mice

C57BL/6 J mice were obtained from the Jackson Laboratory (Bar Harbor, Maine, USA). All experiments were performed with the full knowledge and approval of the Standing Committee on Animals at Boston University in accordance with relevant guidelines and regulations (IACUC protocol number AN15276).

### Cell lines, BMDMs culture

Mouse macrophage cell line J774A.1 was cultured in DMEM/F12 containing 10% fetal bovine serum supplemented with 1% glutamine, penicillin and streptomycin (100 U/mL and 100 μg/mL respectively) and 10 mM Hepes buffer (complete medium). J7-21 cell line generated in our lab by stable transduction of GFP-Ipr1 in J774A.1 cells. Isolation of mouse bone marrow and culture of BMDMs were carried out as previously described^21^. iBMM stably expressing EGFP-LC3(GFP-LC3) was a kind gift from Hardy Cornfield and grown in complete medium. 293TR-FLuc cells that stably express Renilla firefly luciferase was grown in complete medium and was a gift from Luke Whitesell of Whitehead Institute.

### Growth of *F. tularensis* LVS and macrophage infection

*F. tularensis* live vaccine strain (LVS) were grown in Brain-Heart Infusion broth overnight, harvested and then diluted in media without antibiotics to get the desired MOI. BMDM were seeded in tissue culture plates. At time of infection cells were washed with media without antibiotics and infected at indicated MOI. The plates were then centrifuged at 500 × *g* for 15 minutes and incubated for 1 hr at 37 °C with 5% CO_2_. Cells were then washed with fresh media, and incubated for 45 min at 37 °C with media containing gentamicin (50 μg/mL) to kill any extracellular bacteria. Cells were washed again and cultured in DMEM/F12/10% FBS medium without antibiotic at 37 °C in 5% CO_2_.

### Optimization and validation of assay

The macrophage cell line J774 was transduced with lentiviral constructs for Dox- inducible GFP-Ipr1 expression and clones were selected that had negligible basal level of GFP- Ipr1 expression and high inducibility with IFNγ. Using these clones eliminated background and greatly improved the assay sensitivity and reproducibility, crucial factors for the high throughput analyses. One such clone, clone 21 (J7-21), was used in further studies^21^. First, we tested different concentrations of IFNγ (0.05–50 U/mL) and time points (24, 48 hrs) after addition of Dox to determine optimal assay conditions. Celigo Cytometer was used to measure number of GFP-Ipr1 positive cells and GFP-Ipr1 fluorescence intensity per nucleus after counterstaining with nuclear stain DAPI. Fluorescence intensity was maximal after 48 hrs of IFNγ priming and 24 hrs after addition of Dox (optimal dose of Dox has been determined as well). This and several similar optimization experiments were performed in 96-well plate format. J7-21 cells were plated in 96 well plates at 7,000 cells per well, primed with increasing concentrations of IFNγ ranging from 0.05 to −25 U/mL for 48 hrs, Dox was added for the last 24 hrs and the GFP-Ipr1 fluorescent cells were counted using Celigo Cytometer. We observed increase in signal at 0.2 U/mL of IFNγ, while the maximal levels were achieved at 10 U/mL. The dose-response curve demonstrated the high sensitivity and linearity of the assay in the range of 0.2–10 U/mL of IFNγ. Given its wide dynamic range, the assay can, in principle was used to screen for activators of IFNγ priming using 0.2 U/mL of IFNγ. We developed a gating strategy to identify the hits. The fluorescence intensity of GFP-Ipr1 at 0.2 U/mL IFNγ was set as a threshold for our gating and we selected hits based on compounds that showed at least four fold increase in GFP-Ipr1 signal above the background.

### Library screening

Screening was carried out at the high throughput screening facility at Boston University. The ICCB and the LOPAC library stocks were provided by the screening facility. J7-21 cells were seeded at 7000 cells per well in 96 well plates the previous day and compounds were added in the presence of 0.2 U/mL IFNγ. Final compound concentrations in the initial screen were approximately 1 μM. After 24 hrs, 1 μg/mL dox was added and GFP-Ipr1 expression was measured 24 hrs later using celigo cytometer. The wells were scored based on fluorescent intensity of GFP-Ipr1. Interferon-gamma (Peprotech) used at a concentration of 10 U/mL served as a positive control. For all follow-up work, the BU-CMD compounds were resynthesized, validated as referenced in the text. The commercially available compounds from ICCB and LOPAC were purchased from Enzo life sciences.

### Chemical Synthesis

Synthetic compounds were obtained from the chemical collection at the BU Center for Molecular Discovery (BU-CMD). Compounds including C5557, C8808, and C8809 were synthesized using the reported procedures^64^.

### Immunoblotting

To monitor the Ipr1 protein levels we have developed Ipr1 peptide-specific rabbit polyclonal antibodies, which recognized the Ipr1 protein of predicted length on Western blots in nuclear, but not cytoplasmic, extracts of IFNγ-treated J774A.1 cells (Supplementary Fig. S1). J7-21 cells and BMDM’s were subjected to treatments specified in the text. Nuclear extracts were prepared as mentioned previously^21^. Whole cell extracts were prepared by lysing the cells in RIPA buffer supplemented with protease inhibitor cocktail and phosphatase inhibitor I and II (Sigma). Equal amounts (30 μg) of protein from whole-cell extracts was separated by SDS-PAGE and transferred to PVDF membrane (Millipore). After blocking with 5% skim milk in TBS-T buffer [20 mM Tris–HCl (pH 7.5), 150 mM NaCl, and 0.1% Tween20] for 2 hour, the membranes were incubated with the primary antibody overnight at 4 °C. Bands were detected with enhanced chemiluminescence (ECL) kit (Perkin Elmer). Stripping was performed using WB stripping solution (Thermo scientific). The loading control β-actin (Sigma, 1:2000) was evaluated on the same membrane. The Ipr1-specific rabbit anti-serum was generated by Covance Research Products, Inc. (Denver, CO, USA). (1:500) and described previously^21^. The Ipr1 monoclonal antibodies were generated using Ipr1 peptides from Abmart. LC3B antibodies were obtained from Cell signaling(1:250). LC3 II/LC3 I ratio was calculated for each blot by densitometric analysis. IRF1 antibody was obtained from Cell Signalling (1:500). Fold induction of IRF1 was calculated relative to untreated (set as 1) by densitometric analysis after normalizing it to loading control β-actin.

### RNA Isolation and quantitative PCR

Total RNA was isolated using the RNeasy Plus mini kit (Qiagen). cDNA synthesis was performed using the SuperScript II (Invitrogen). Real-time PCR was performed with the GoTaq qPCR Mastermix (Promega) using the CFX-90 real-time PCR System (Bio-Rad). Oligonucleotide primers were designed using Primer 3 software (Supplementary Table S2) and specificity was confirmed by melting curve analysis. Thermal cycling parameters involved 40 cycles under the following conditions: 95 °C for 2 mins, 95 °C for 15 s and 60 °C for 30 s. Each sample was set up in triplicate and normalized to RPS17 or 18 S expression by the DDCt method.

### Immunofluorescence microscopy

Cells were fixed with 4% paraformaldehyde for 15 min at room temperature, permeabilised with 0.25% Triton-X for 30 min and then blocked for 20 min with goat-serum (2.5%). Cells were incubated with primary antibodies [mouse monoclonal antibodies against Ipr1(1:2000), iNOS(1:200) and LC3B(1:250)] overnight at 4 °C in 2.5% goat serum, and incubated with Alexa Fluor 594-conjugated donkey anti-mouse IgG (excitation/emission maxima ~ 590/617 nm) or Alexa Fluor 488- conjugated donkey anti-mouse IgG (excitation/emission maxima ~ 490/525 nm) (1:1000, Invitrogen) secondary antibody for 2 hrs. *F.t.* LVS were detected with anti-mouse *F.t.* LVS antibodies (1:1000). BMDMs from C57BL/6 J mice were grown in coverslips and treated with compounds with indicated time and autophagy was detected using the cyto-ID Autophagy detection kit (Enzo lifesciences) using FITC channel. Images were taken immediately in Nikon-deconvolution microscope. To detect autophagy in immortalized GFP-LC3^ + ^(ex/emi max ~488/510 nm), iBMM was grown as mentioned previously^65^ and samples were processed as mentioned above. Images were acquired using Leica SP5 confocal microscope. All images were processed using Image J software.

### Measurement of nitrite concentration

To measure nitrite (NO_2_^−^), 50 μL of macrophage culture supernatant was collected, mixed with an equal volume of Griess reagent (1% sulfanilamide/0.1% *N*-(1-naphthyl)-ethylenediamine dihydrochloride/2.5% H_3_PO_4_) and incubated for 10 min at room temperature. Nitrite concentration was determined by measuring the absorbance at 540 nm.

### Hoechst/PI Staining Method for cell cytotoxicity

For cell viability assays J7-21 cells or BMDM were plated in 96 well tissue culture plates (12000 cells/well) and subjected to necessary treatments. The supernatant was aspirated and to each well 100 μl PBS containing Hoechst (Invitrogen, 10 μM) and PI (Calbiochem, 2 μM) were added. The plates were kept at 37 °C for 15 min and read in an automated cell cytometer. The % of total and dead cells was calculated for each treatment.

### Reporter assay for measuring translation inhibition

293TR-Fluc cells were grown to confluence and maintained in complete medium. 10,000 cells were plated in a 96-well clear bottom plate in complete medium without phenol-red. Compounds were added at different dilutions and kept for 18 hrs. The next day the media was removed and 100 ul of Nanolight Firefly Luc Assay reagent was added to the wells and the luminescence was measured using a Tecan-plate reader after 2 mins. Cells treated with DMSO served as negative controls and with 100 μg/mL cycloheximide served as positive controls. Percentage of translation inhibition for each compound was calculated in triplicates from two independent experiments.

## Additional Information

**How to cite this article**: Bhattacharya, B. *et al.* Fine-tuning of macrophage activation using synthetic rocaglate derivatives. *Sci. Rep.* **6**, 24409; doi: 10.1038/srep24409 (2016).

## Supplementary Material

Supplementary Information

Supplementary Table S1
