# Supplementary material for: Fine-tuning of macrophage activation using synthetic rocaglate derivatives
Source: Sci Rep. 2016 Apr 18;6:24409. doi: 10.1038/srep24409 (PMC4834551; doi:10.1038/srep24409)
Supplement: Supplementary Information [file srep24409-s1.pdf]

Supplementary Information

Fine tuning of macrophage activation using synthetic rocaglate derivatives

Bidisha Bhattacharya[1], Sujoy Chatterjee[1], William G. Devine[2], Lester Kobzik[3]
Aaron B. Beeler[2], John A. Porco, Jr.[2] and Igor Kramnik[1*]

[1] Pulmonary Center, Department of Medicine, Boston University School of Medicine, National Emerging Infectious Diseases Laboratories (NEIDL), Boston University, Boston, MA, 02118, USA.

[2]Department of Chemistry, Center for Molecular Discovery (BU-CMD), Boston University, Boston, MA, 02215, USA.

[3]Department of Environmental Health, Harvard School of Public Health, Boston, MA, 02115, USA.

[*]Correspondence and requests for materials should be addressed to I.K.(email: ikramnik@bu.edu).

Supplementary Methods:

Microarray analysis:

BMDM from C57BL/6 mice were treated with 0.2U/ml IFNγ in the presence and absence of 1 μM C9433 for 24hrs. Total RNA was isolated using RNeasy Plus Mini kit(Qiagen) according to manufacturer's instructions. Biotin labeling was performed using the Ambion WT Expression Kit (Life Technologies, Grand Island, NY) according to the manufacturer's protocol, followed by the GeneChip WT Terminal Labeling and Controls Kit (Affymetrix, Santa Clara, CA). The labeled, fragmented DNA was hybridized to the Affymetrix GeneChip Mouse Gene 2.0 ST Array for 18 hours in a GeneChip Hybridization oven 640 at 45°C with rotation (60 rpm). The hybridized samples were washed and stained using an Affymetrix fluidics station 450 with streptavidin-R-phycoerythrin (SAPE) and the signal was amplified using a biotinylated goat anti-streptavidin antibody followed by another SAPE staining (Affymetrix Hybridization, Washing and Staining Kit). After staining, microarrays were immediately scanned using an Affymetrix GeneArray Scanner 3000 7G Plus. CEL files were normalized to produce gene-level expression values using the implementation of the Robust Multiarray Average (RMA) in the affy package (version 1.36.1) included within in the Bioconductor software suite (version 2.12) and an Entrez Gene-specific probeset mapping (version 17.0.0) from the Molecular and Behavioral Neuroscience Institute (Brainarray) at the University of Michigan. Array quality was assessed by computing Relative Log Expression (RLE) and Normalized Unscaled Standard Error (NUSE) using the affyPLM Bioconductor package (version 1.34.0). Analyses of variance were performed using the f.pvalue function in the sva package (version 3.4.0). Pairwise differential gene expression was assessed by performing Student's t test on the coefficients of simple linear models computed using the lmFit function in the limma package (version 3.14.4). Correction for multiple

hypothesis testing was accomplished using the Benjamini-Hochberg false discovery rate (FDR). Human homologs of mouse genes were identified using HomoloGene (version 67). All microarray analyses were performed using the R environment for statistical computing (version 2.15.1).

Gene Set Enrichment Analysis (GSEA)

GSEA (version 2.0.13) was used to identify biological terms, pathways and processes that were coordinately up- or down-regulated within each pairwise comparison. The Entrez Gene identifiers of the human homologs of the genes interrogated by the array were ranked according to the t statistic computed for the C2 versus vehicle comparison within each cytokine group. Mouse genes without a human homolog were removed, and t statistics for multiple mouse genes with the same human homolog were averaged prior to ranking. The resulting ranked lists were each used to perform a pre-ranked GSEA analysis (default parameters with random seed 1234) using the Entrez Gene versions of the Biocarta, KEGG, Reactome, Gene Ontology (GO), and transcription factor and microRNA motif gene sets obtained from the Molecular Signatures Database (MSigDB), version 4.0.

Chromatin Segregation Assay

J7-21 cells (3x 10^7) were washed with PBS and once with 1 ml hypotonic lysis buffer. Pelleted cells were resuspended in hypotonic lysis buffer [10 mM HEPES pH 7.9, 10 mM KCl, 1.5 mM $MgCl_2$, 0.2% NP40], containing protease inhibitor cocktail and phosphatase inhibitor cocktail I and II (Sigma) and was incubated on ice for 15 min. Cell lysates were centrifuged for 3 min at 1,000 x g, 4°C (cytosolic fraction). The pelleted nuclei was gently resuspended (by tapping, but not pipetting, to prevent chromatin decondensation) in high-salt buffer (10 mM HEPES with pH 7.9, 420 mM NaCl, 1.5 mM $MgCl_2$, 25%

glycerol, EDTA-free complete protease inhibitor cocktail) and incubated on ice for 1 hr. Samples were centrifuged for 10 min at 10,000 rpm (nucleoplasmic fraction). The pellet was then resuspended in nuclease buffer (10 mM HEPES with pH 7.9, 300 mM NaCl, 2.5 mM $CaCl_{2,}$ 20 mM KCl, 2% glycerol, 0.2% Triton X-100, EDTA-free complete protease and phosphatase inhibitor cocktail) containing 100 U/ml micrococcal nuclease (Takara) and incubated at $4^{o}C$ for 1 hour. Chromatin digestion was stopped by adding EDTA to a final concentration of 5 mM and samples were vortexed briefly and centrifuged for 15 min at 14,000 rpm (chromatin fraction).

IP of GFP-Ipr1-Containing Molecular Complexes

J7-21 cells were seeded in four 75cm tissue-culture plates and treated with doxycycline (1 μg/ml) and IFNγ (100U/ml) for 24 hrs. For kinetics experiments cells treated with dox and IFNγ were harvested at 0, 6, 12 and 24hrs. Cells were lysed with a hypotonic lysis buffer (10 mM HEPES pH 7.9, 10 mM KCl, 1.5 mM $MgCl_2$, 0.2% NP40, protease inhibitor cocktail and phosphatase inhibitor cocktail I and II (Sigma), and were incubated on ice for 15 min with occasional vortexing, transferred to microfuge tubes and spun at 4°C, 14,000 rpm for 3 min. The liquid fraction containing cytosolic proteins was discarded, and nucleoplasmic and chromatin fractions were prepared as mentioned above. Approximately 400 μg of nucleoplasmic and chromatin supernatants were added to 25 μl Protein G magnetic beads covalently conjugated to GFP-specific mAb (Miltenyi Biotec) and/or isotype specific IgG (Abcam). Samples were incubated for 4 hr with rotation at 4°C. Beads were washed (3x) with lysis buffer containing 0.05% NP40, and once with ice-cold TE buffer. Bound proteins were eluted by boiling for 5 min in sample buffer, and the eluted samples (∼30μl) were loaded on a 12% SDS-PAGE and separated. Immunoblotting was carried out and probed with H3K9Me3 antibodies (Active motif 1:1000).

Supplementary Figure S1: Nuclear localization and chromatin association of Ipr1

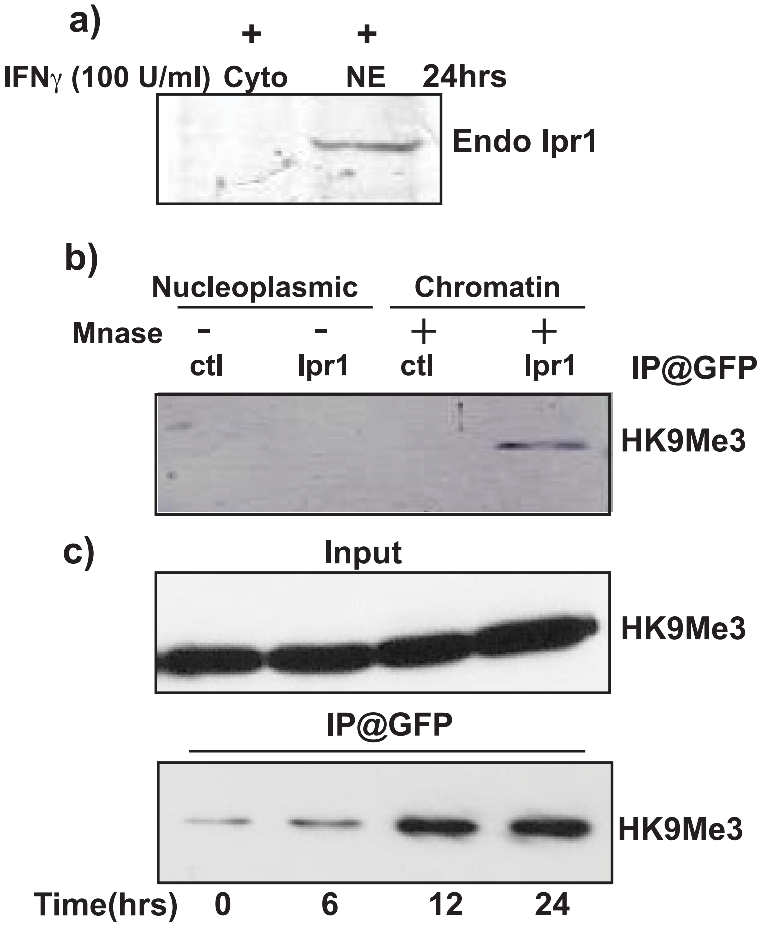

Supplementary Figure S2: Ipr1 undergoes processing and is subjected to post-translational modification in the nucleus of activated macrophages

Predicted phosphorylation sites for Ipr1

a)

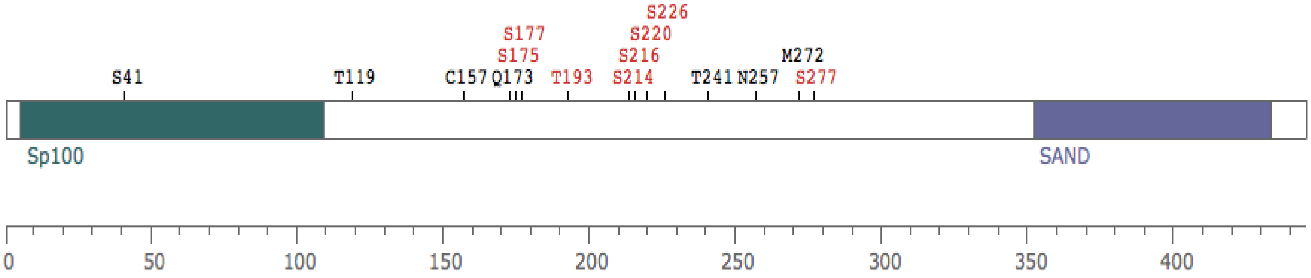

Predicted cleavage sites for Ipr1

b)

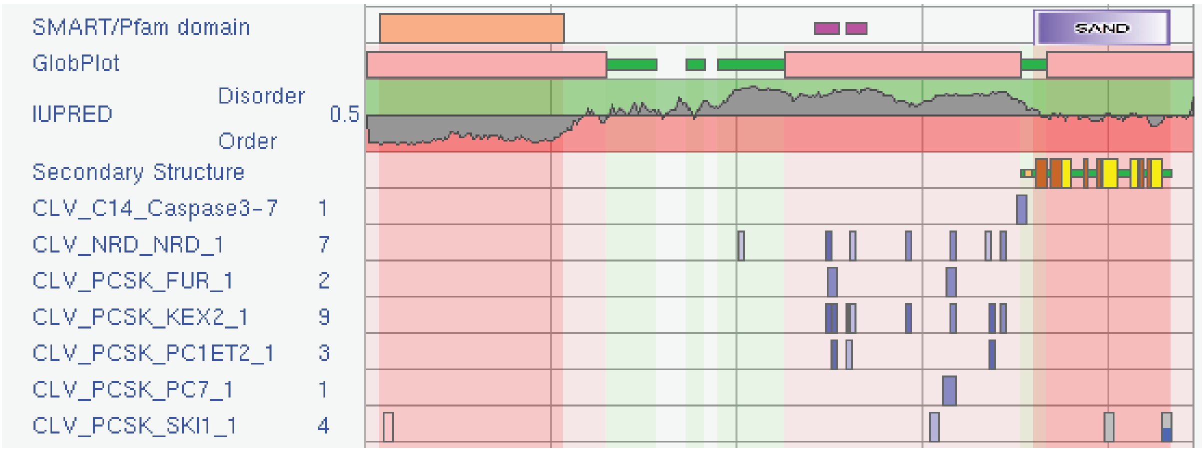

c)

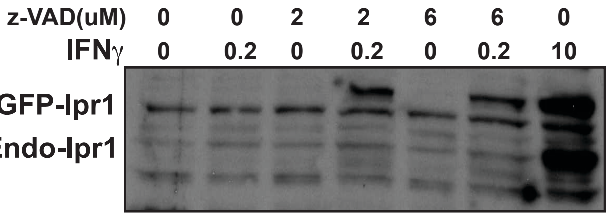

d)

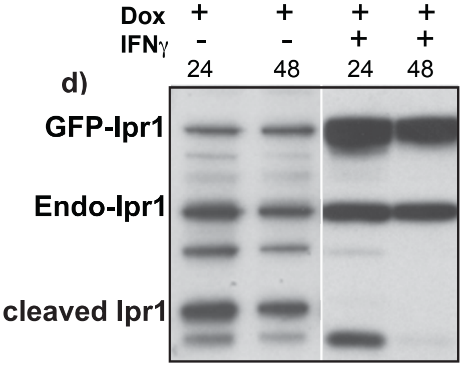

e)

Time	GFP-Ipr1	Endo-Ipr1	Cleaved product	Ratio of cleaved Ipr1 vs full-length Ipr1(endo+GFP)
6hr	788.062	2636.569	463.506	0.135
12hr	4622.619	5116.79	526.749	0.054
24hr	7214.033	9747.983	8381.832	0.494
48hr	7743.497	8474.74	7985.539	0.492

Supplementary Figure S3: Synthesis of CMLD009433

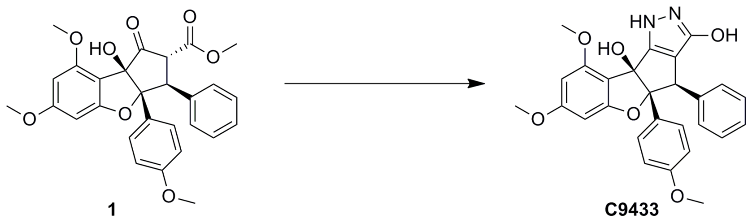

Supplementary Figure S4: iNOS inhibition by rocaglates C9433 and C8809

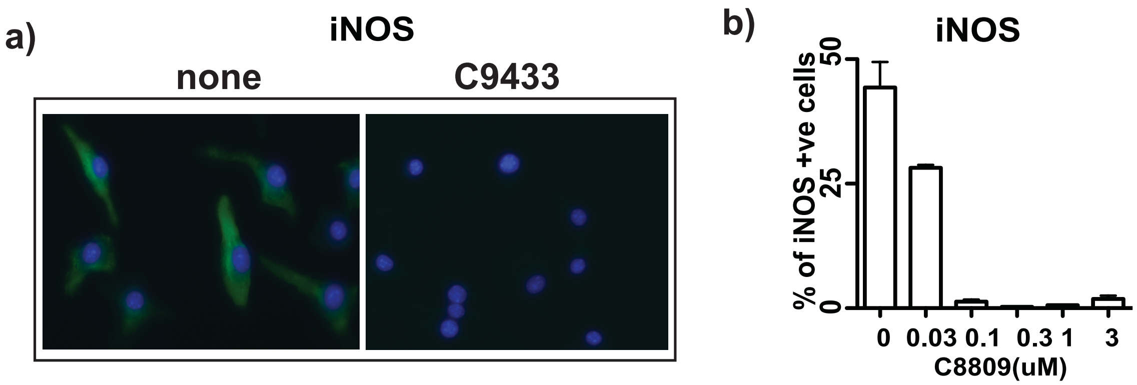

Supplementary Figure S5: Gene expression of new rocaglates

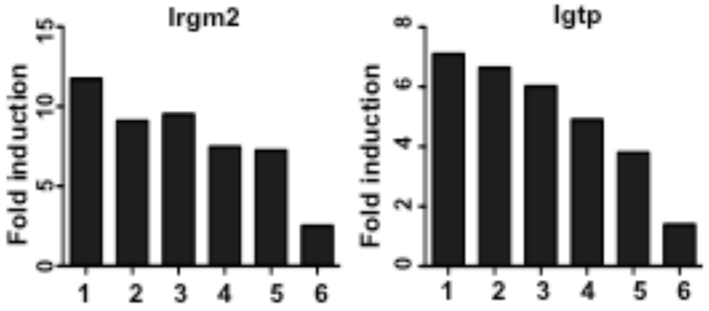

Supplementary Figure S6: Characterisation of rocaglates C8808 and C5557

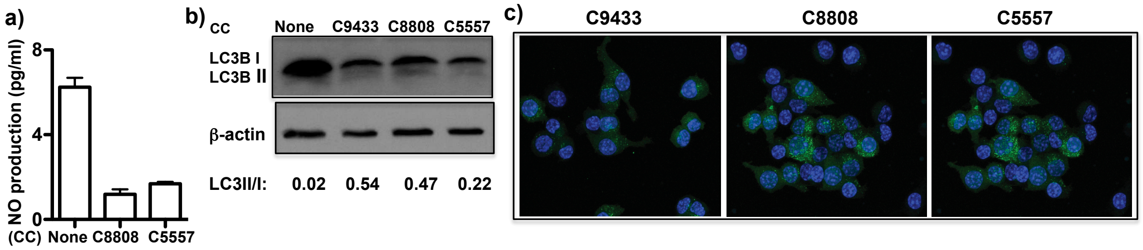

Supplementary Figure S7: Chemical structure of RHT and its enantiomers

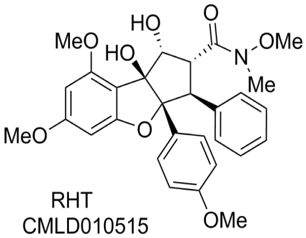

RHT
CMLD010515

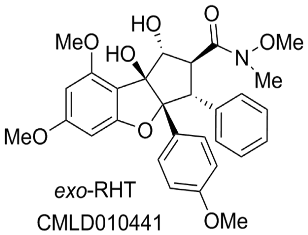

exo-RHT
CMLD010441

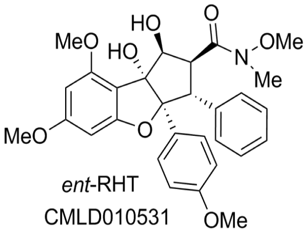

ent-RHT
CMLD010531

Supplementary Table S2: Primers used for real-time PCR amplification

Ido1	ACTGTGTCCTGGCAAACTGGAAG	AAGCTGCGATTTCCACCAATAGAG
Igtp	GAAAGAAGCCGAGGACCAC	TCTGTCACCGCCTTACCAAT
Irgm1	TGGCAATGGCATGTCATCTT	AGTACTCAGTCCGCGTCTTCGT
Irgm2	CGGAGAGCTGTGGAGAGAGA	GCCATGTTTATGAAAAGTGTAAAAGT
Irf5	AATACCCCACCACCTTTTGA	TTGAGATCCGGGTTTGAGAT
Irf7	CTGGAGCCATGGGTATGCA	AAGCACAAGCCGAGACTGCT
Ipr1	ACACTCCCTGTGACCTGTGG	GCCAATCTCCTGCCTCATTC
Irf1	CAGAGGAAAGAGAGAAAGTCC	CACACGGTGACAGTGCTGG
18S	TCAAGAACGAAAGTCGGAGGT	CGGGTCATGGGAATAACG
Ptgs2	TCTCCAACCTCTCCTACTAC	ACTCTCTCCGTAGAAGAACC
Gadd45b	TGTACGAGGCGGCCAAACTG	TGTCGCAGCAGAACGACTGG
IFNβ	ATGAGTGGTGGTTGCAGGC	TGACCTTTCAAATGCAGTAGATTC
IP10	GACGGGTCCGCTGCAACT	GCTTCCCTATGGCCCTCATT
IL10	CCAGTACAGCCGGGAAGACAATA	TGGCAACCCAAGTAACCCTTAAA
RPS17	TGTCGGGATCCACCTCAATG	CGCCATTATCCCCAGCAAG

Supplementary Figure legends

Suppl Fig S1: Ipr1 associates with the nucleus of activated macrophages. a) J7-21cells were treated with 100 U/ml IFNγ for 24hrs and fractionated in nuclear and cytoplasmic fractions. Endogenous Ipr1 was detected in these fractions by immunoblotting using Ipr1specific rabbit polyclonal antibodies. NE and Cyto denote nuclear extracts and cytoplasmic extracts respectively. b) Validation of chromatin associated GFP-Ipr1 partners. J7-21 cells were treated with dox and IFNγ for 24hrs and separated into nucleoplasmic and chromatin fractions. IP was performed with nucleoplasmic and chromatin fractions using anti-GFP monoclonal antibody conjugated to magnetic beads. Immunoblotting was carried out using H3K9me3 antibodies. c) Kinetics of association of H3K9Me3 with GFP-Ipr1. J7-21 cells were treated with dox and IFNγ for 0, 6, 12 and 24hrs and IP was carried out with the chromatin fraction. Blots were probed with H3K9Me3. Input refers to the chromatin fraction before immunoprecipitation.

Supplementary Fig S2: Ipr1 is subjected to posttranslational modification and processing in activated macrophages. a)The predicted phosphorylation sites of Ipr1 were generated using PhosphoSitePlus (www.phosphosite.org). b) Ipr1 undergoes proteolytic cleavage. The predicted cleavage sites for Ipr1 was generated using the Eukaryotic Linear Motif (ELM) resource for Functional Sites in Proteins. c) Immunoblot analysis of whole-cell extracts of J7-21 cells treated with 2 and 6μM z-VAD in presence and absence of 0.2U/mL IFNγ and 1μg/mL dox for 24hrs. Cells treated with 10 U/ml IFNγ was used as positive control. Blots were probed with Ipr1 polyclonal antibodies. d) J7-21 cells were treated with 10 U/mL IFNγ and 1μg/mL dox for 24hrs and 48hrs. Immunoblot was carried out with whole cell extracts and probed with Ipr1 polyclonal antibodies. e) The ratio of cleaved vs total Ipr1(endogenous Ipr1 and GFP-Ipr1 from Fig. 1b was calculated by densitometry using Image J software.

Supplementary Fig S3: Synthesis of CMLD009433 (C9433): To a 10 mL microwave vial equipped with a stir bar was charged methyl keto-rocaglate **1** (32 mg, 0.07 mmol), toluene (1.4

mL), and hydrazine monohydrate (34 µL, 0.70 mmol). The vial was sealed with a septum and the contents were irradiated to 140 °C in a microwave reactor for 30 minutes. The mixture was then cooled to room temperature, poured over water (10 mL), quenched with sat. aq. $NaHCO_3$ (3 mL), and finally extracted with ethyl acetate (3 x 12 mL). The combined organic layers were washed with brine (5 mL), dried over Na_2SO_4, and concentrated. The residue was purified by flash column chromatography using a gradient of 1-10% CH_3OH in CH_2Cl_2. Hydroxypyrazolo rocaglate C9433 (11.6 mg, 0.025 mmol) was collected as a light beige solid in 35% yield. **TLC** (5% CH_3OH/CH_2Cl_2) R_f = 0.26. **mp**: decomposed. **IR** vmax (film) 3394 (br.), 2963, 2923, 2854, 1614, 1513, 1463, 1249, 1219, 1201, 1179, 1148, 1112, 1040, 1018. **^{1}H NMR** (500 MHz, DMSO-d_6) δ ppm 11.72 (br. s., 1 H), 9.58 (br. s., 1 H), 7.02 - 7.11 (m, 4 H), 6.94 - 7.01 (m, 3 H), 6.53 (d, *J*=8.8 Hz, 2 H), 6.38 (d, *J*=2.0 Hz, 1 H), 6.14 (d, *J*=2.0 Hz, 1 H), 5.41 (s, 1 H), 4.22 (s, 1 H), 3.76 - 3.81 (m, 6 H), 3.57 (s, 3 H). (500 MHz, acetone d_6) δ ppm 7.24 (d, *J*=7.3 Hz, 2 H), 7.14 (d, *J*=8.8 Hz, 2 H), 7.08 (t, *J*=7.6 Hz, 2 H), 6.99 (t, *J*=7.3 Hz, 1 H), 6.56 (d, *J*=8.8 Hz, 2 H), 6.32 - 6.36 (m, 1 H), 6.12 - 6.16 (m, 1 H), 4.45 (s, 1 H), 3.83 - 3.85 (m, 3 H), 3.80 - 3.83 (m, 3 H), 3.60 - 3.64 (m, 3 H). **^{13}C NMR** (500 MHz, DMSO-d_6) δ ppm 162.9, 159.7, 157.5, 157.3, 138.5, 128.6, 128.4, 127.7, 127.2, 125.8, 112.4, 111.7, 111.7, 108.5, 92.1, 89.1, 83.1, 55.6, 55.6, 55.3, 54.7, 54.7, 54.3. (500 MHz, acetone-d_6) δ ppm 164.8, 161.4, 159.3, 158.8, 156.9, 151.2, 139.6, 130.0, 129.3, 129.0, 128.2, 126.8, 113.9, 112.8, 109.2, 106.4, 93.0, 90.0, 84.6, 56.1, 55.9, 55.8, 55.2. **LC/MS** found 473.4 $[M+H]^+$. **HRMS**: *m/z* calcd for $[C_{27}H_{24}N_2O_6+H]^+$ 473.1713, found 473.1719 (1.27 ppm).

Supplementary Fig S4: Rocaglates C9433 and 8809 suppresses iNOS expression in primary macrophages. a) Microscopy of BMDM treated with 15 ng/mlTNFa and 10 U/ml IFNg for 24hrs in the presence and absence of C9433 to detect iNOS protein expression. b) iNOS expression was detected in BMDM treated above in the presence of C8809 using specific anti-iNOS antibody and the fluorescence intensity was measured using celigo. All measurements for iNOS expression were performed in triplicates.

Supplementary Fig S5: Comparative gene expression of new rocaglates. BMDM were treated with 0.33 µM compound (1-C9433, 2-C8809, 3-C10021, 4-C7564, 5-

C7565, and 6-C10361) for 24hrs and mRNA expression of Igtp and Irgm2 was measured by qPCR. Gene expression was normalized to expression of 18S and presented relative to expression in 0.2U IFNγ treated cells (set as 1).

Suppl Fig S6: Rocaglates C8808 and C5557 induces autophagy and suppresses NO production in primary macrophages. a) NO production by rocaglates C8808 and C5557. BMDM were treated with 15ng/mL TNFα and 10U/mL IFNγ for 24hrs in the presence and absence of 1 μM compounds and production of NO (assayed as NO_2^-) was determined. All measurements for NO production were performed in triplicates. b) C8808 and C5557 induces autophagy in primary macrophages. BMDM were treated with 1μM compounds for 6 hrs and autophagy was determined by increase of LC3B-II to LC3B-I ratio by immunoblotting. Blots represent data of two independent experiments. c) BMDM was treated with 1μM compounds for 16hrs and autophagy induction was determined using Cyto-ID Autophagy Detection kit. Images represent data from at least two independent experiments

Suppl Fig S7: Chemical structure of RHT and its stereoisomers
